# BIOPEP-UWM Database of Bioactive Peptides: Current Opportunities

**DOI:** 10.3390/ijms20235978

**Published:** 2019-11-27

**Authors:** Piotr Minkiewicz, Anna Iwaniak, Małgorzata Darewicz

**Affiliations:** Chair of Food Biochemistry, University of Warmia and Mazury in Olsztyn, Plac Cieszyński 1, 10-726 Olsztyn-Kortowo, Poland; ami@uwm.edu.pl (A.I.); darewicz@uwm.edu.pl (M.D.)

**Keywords:** bioactive peptides, database, proteolysis, SMILES code, foods, nutrition, chronic diseases, nutraceuticals

## Abstract

The BIOPEP-UWM™ database of bioactive peptides (formerly BIOPEP) has recently become a popular tool in the research on bioactive peptides, especially on these derived from foods and being constituents of diets that prevent development of chronic diseases. The database is continuously updated and modified. The addition of new peptides and the introduction of new information about the existing ones (e.g., chemical codes and references to other databases) is in progress. New opportunities include the possibility of annotating peptides containing D-enantiomers of amino acids, batch processing option, converting amino acid sequences into SMILES code, new quantitative parameters characterizing the presence of bioactive fragments in protein sequences, and finding proteinases that release particular peptides.

## 1. Introduction

The BIOPEP-UWM database is freely-accessible without registration at the following website: http://www.uwm.edu.pl/biochemia/index.php/pl/biopep. Recently, bioinformatic databases and software represent basic tools in the research on biologically active peptides, e.g., those derived from food. Their role was described in several reviews [1,2,3,4,5,6,7]. The BIOPEP-UWM™ (formerly BIOPEP) database of bioactive peptides is one of these tools. It has been available on the internet since 2003. Its previous versions have been described in publications by Minkiewicz et al. [8] and Iwaniak et al. [9]. The database has recently been widely used in food and nutrition science as a source of information about peptides being in the focus of interest as putative components of functional foods involved in the prevention of chronic diseases [5,7,10]. Over 350 articles are available that describe results that had been obtained, verified, or interpreted with the help of the BIOPEP-UWM database of bioactive peptides (excluding these contributed by database curators). Links to the BIOPEP-UWM™ database are recently available via such websites as MetaComBio [11], LabWorm, and OmicX. Information about peptides from the database is integrated into the SpirPep [12] and FeptideDB [13] databases.

The BIOPEP-UWM™ database is continuously updated and modified. Several new options have been introduced since the publication of the last article describing it [9]. The aim of the present publication is to provide information helpful in work with the current version of the database and associated tools, including the use of new options introduced in the last three years.

## 2. Database Organization 

**Figure 1:**
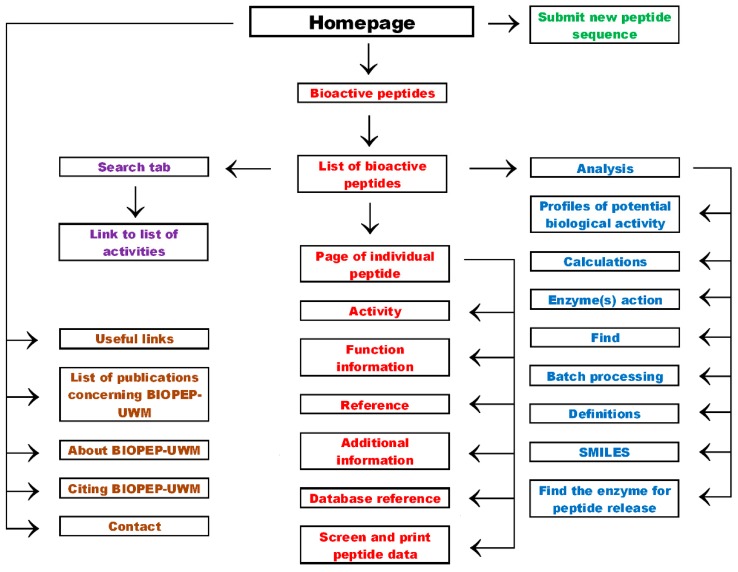
Scheme of organization of the BIOPEP-UWM database of bioactive peptides.

The scheme of organization of the BIOPEP-UWM homepage is presented in Figure 1. The screenshot of the homepage is available in Supplementary Figure S1. Apart from a database of bioactive peptides described in this article, the BIOPEP-UWM contains databases of proteins, allergenic proteins, and their epitopes [14] as well as sensory peptides and amino acids [9]. The homepage also has a tab that allows users to submit new peptide sequences (not annotated yet in the database) or new activities (not annotated) of the existing peptides (See Supplementary Figure S2), and also a new BIOPEP-UWM news tab (not indicated in Figure 1). 

The “bioactive peptides” tab links with the list of bioactive peptides (Supplementary Figure S3). Access to more detailed information about a particular peptide sequence is available via the “peptide data” tab attributed to each peptide. The page with a peptide list contains links to associated tools enabling the processing of peptide and protein sequences (via the “analysis” tab). Scrolling down using the bar left from the table (Supplementary Figure S3) opens the window, which allows the input of queries, enabling a search.

## 3. Enlarging the Number of Peptides in the Database by BIOPEP-UWM™ Users 

The BIOPEP-UWM database is a curated database. Although it is regularly enriched with the new peptides, it is rather impossible to insert all bioactive peptides that are continuously being found in the literature. Thus, the “submit new peptides” option (see the BIOPEP-UWM homepage; Supplementary Figure S1) enables users to send us a peptide sequence not found in our database so far. The peptide sequence to be added to BIOPEP-UWM has to be provided in a one-letter code by pasting it to the window that appears after clicking the “submit new peptides” tab. All peptides sent this way are verified by our curators and can be uploaded to the database on condition that the sender had provided e-mail and reference data (i.e., details of an article the peptide was published in). Providing the senders’ address enables generating an automatic e-mail confirming that the peptide of interest was successfully submitted by the user to the BIOPEP-UWM database. Publication details are needed to verify the information sent. The lack of the sender’s e-mail as well as reference data on peptide to be inserted to BIOPEP-UWM (mandatory fields for successful submission) makes the submitted information incomplete and may temporarily eliminate the sequence from the process of uploading it to our database.

## 4. Peptide Information 

The current layout of peptide information in the BIOPEP-UWM database has earlier been used in the database of sensory peptides and amino acids [9]. Its implementation into the database of bioactive peptides is still in progress. Information about an example peptide with a GHS sequence (BIOPEP-UWM ID 9473) [15] is presented in Table 1. The screenshot of a peptide page is presented in Figure S4.

The ID number is the first piece of information displayed on a peptide page. A peptide with a single activity annotated in the BIOPEP-UWM possesses one ID number. A peptide annotated as multifunctional possesses more ID numbers. The representative GHS peptide is annotated in the BIOPEP-UWM database twice, i.e., as an inhibitor of renin (EC 3.4.23.15) and angiotensin-converting enzyme (EC 3.4.15.1) [15] (ID 9472 and 9473, respectively). Database ID may serve as an unambiguous identifier of a compound, e.g., peptide. Examples of using ID numbers from peptide databases (e.g., BIOPEP-UWM) as peptide identifiers may be found in, e.g., recent publications of Skrzypczak et al. [16] and Khazaei et al. [17].

Name is the second piece of information on the page of an individual compound. Peptide names are often identical to their activity (e.g., ACE inhibitor). Some well-known peptides possess their own names, e.g., soybean lunasin (BIOPEP-UWM ID 9525 and 9526), the role of which has been reviewed by Hsieh et al. [18].

Peptide sequences are annotated in the BIOPEP-UWM database of bioactive peptides using a standard one-letter code describing 20 protein amino acids and their d-enantiomers (a recently added option). A peptide with the FhL sequence (l-Phe-d-His-l-Leu) [19] (BIOPEP-UWM ID 9475) may serve as an example of the peptide containing D-amino acid residue. The database offers an opportunity to annotate C-terminal amidation using the “~” symbol [8]. This symbol is, however, not universal. The EROP-Moscow database [20] uses the “z” symbol for the same purpose. There is also an opportunity (not exploited to date) to annotate phosphoserine using “B” and “b” symbols for l- and d-enantiomer, respectively. InChIKey is an unambiguous chemical identifier [21]. It always contains 27 characters and is sufficient for search via both search engines of chemical databases and common search engines such as Google^TM^. InChIKey is used as a name in the case of some compounds annotated in the PubChem database [22].

Information about the biological activity is inserted as activity (short version), activity code (abbreviation of activity), and function (more detailed version). The current list of activities of peptides found in the BIOPEP-UWM database of bioactive peptides is provided in Table 2. The list of bioactivities has been rearranged as compared to this published in 2008 [8] to remove redundancy (e.g., remove synonymous or extremely rare activities). On the other hand, several new activities, especially these concerning inhibition of enzymes, have recently been added. Annotation of bioactive peptides as compounds interacting with individual enzymes is preferred by users of the BIOPEP-UWM database, as in the case of, e.g., renin inhibitors [23,24,25]. Information concerning the role particular enzymes play in metabolic pathways has recently become available in specialized databases [26].

The peptide entry page also provides the chemical (average) and monoisotopic molecular mass of the peptide and a reference describing its given activity.

Completion of the contents of “additional information” and “database references” tabs is in progress. The “additional information” tab includes peptide structure written using chemical codes called SMILES [27]—the most popular chemical code, and InChI—recommended by IUPAC [21]. These codes represent a typical language of cheminformatics (i.e., chemical informatics) [26]. Cheminformatics is considered as an emerging method in food science [28,29]. SMILES and InChI codes, as well as InChIKeys, are used as input data for the search of molecules in chemical databases [26,30]. The supplement to our previous review [31] may provide insights on how much information about peptide bioactivity is presented in chemical databases. InChIKey is sufficient to search via common search engines such as Google^TM^. This option enables, e.g., finding peptides annotated in the BIOPEP-UWM database. There are many types of software that enable predicting the physicochemical and biological properties of chemical compounds and using, e.g., SMILES. This code may be converted into more than one hundred formats used in chemical informatics, for instance, by OpenBabel software [32]. Examples of using programs which require chemical codes as input data for in silico analysis and prediction of properties of food peptides have been recently presented by Ortiz-Martinez et al. [33], Mojica et al. [34], and Yu et al. [35]. Amino acid sequences are converted into SMILES code using applications available in the BIOPEP-UWM database via the “analysis” tab. Conversion of SMILES representations into InChI and InChIKeys is performed using OpenBabel or MarvinSketch software.

A peptide with a C-terminal amide group cannot be found in protein sequences. Precursors of these peptides, containing C-terminal glycine residues, are thus added. The mechanism of amidation includes the substitution of C-terminal glycine with an amide group [36]. A peptide with ID 2580 may serve as an example of this type of annotation. It is a precursor of antibacterial peptide [37] annotated as ID 2579. Information about amidation is provided in the “additional information” tab of a peptide, being a precursor of the amidated form (in the above example, peptide annotated as ID 2580).

The “additional information” tab also contains brief information about activities of the peptide taken from the BIOPEP-UWM database of bioactive peptides and other databases as well as information about peptide taste from the BIOPEP-UWM database of sensory peptides and amino acids [9].

Information about food resources and products, different values of IC_50_ are also included in the “additional information” tab for some of the peptides.

The “database reference” summarizes databases providing information about a given peptide (for example, see Table 1). The list of databases most commonly cited in the above tab is presented in Table 3. The list has been significantly enriched since the publication of our previous article describing the database [9]. ID numbers of peptides are also provided in particular databases. Some databases (such as ACToR [43] or ChemIDPlus [44]) use CAS registry numbers as compound identifiers. The databases are available via the MetaComBio website [11] or the “useful links” tab on the BIOPEP-UWM website. The list of databases cited has been significantly enlarged since 2016 (Table 3).

The last tab “screen and print peptide data” summarizes all data concerning a given peptide. Supplementary Table S1 is copied directly from the above tab. In the supplement to our previous publication [9], we have pointed out the opportunity for providing links to this tab from other resources. Examples of such links are available in the supplement to our review concerning taste-affecting peptides [31]. Here we offer the opportunity to construct links to peptide pages (“activity” tabs). The data of example peptide (ID 9473) can be found at the following address: http://www.uwm.edu.pl/biochemia/biopep/peptide_data_page1.php?zm_ID=9473. ID at the end of the address (ID = 9473) may be replaced by another one to generate a link to another peptide data. An analogous link to a representative sensory peptide is as follows: http://www.uwm.edu.pl/biochemia/biopep/sensory_data_page1.php?zm_ID=2. 

## 5. Search Options

Search options are summarized in Table 4 and Supplementary Figure S5.

Search options available in the BIOPEP-UWM database of bioactive peptides fall into the following major categories: text-based (ID, name, activity, reference, and InChIKey), structure-based (sequence-based), and property-based (number of amino acid residues and molecular mass). They are typical of peptide databases [4]. The use of an ID number as a query is the first search option. A single ID number corresponds to a single peptide with one defined activity. Search by name or by activity offers two possibilities to the user: finding all names or all activities including the chosen word or text fragment or exact search (see Supplementary Figure S5). The first opportunity leads to finding more peptides that fulfill the search criterion. Using the word “hemorphin-7” as a query, we can find four peptides (ID 2570, 2973, 3079, and 9001) without using the exact search option and only one (ID 3079) using the exact search option (search performed on 30 August 2019). 

The search menu contains a link to the list of activities (Supplementary Figure S5), which serve for a query choice. In contrast to Table 2, the bioactivities are listed in the chronological (not alphabetical) order. Again, it is possible to use the exact search option. Using the word “inhibitor” as a query without using the exact search option has given a list of 1552 peptides as an output (30 August 2019). The list contains all inhibitors of enzymes (e.g., ACE, dipeptidyl peptidase IV, and dipeptidyl peptidase III). The exact search option with the same query found only 67 peptides with the activity annotated as “inhibitor” (see Table 2).

InChIKey is the most typical identifier of compounds (e.g., peptides) in chemical databases (e.g., PubChem [22]; ChemSpider [54], and ChEMBL [40]). Although it is a unique identifier of any chemical compound, it does not provide information about its structure [21]. InChIKeys in the BIOPEP-UWM database correspond to linear peptides with all chirality centers defined, acidic and basic groups electrically neutral, and cysteine residues reduced (if any in the peptide sequence). Incomplete InChIKey used as a query may result in finding more peptides. For instance, a “DYKIIFRCSA-N” fragment occurs in three InChIKeys corresponding to the celiac toxic peptide with the sequence PSQQQP (ID 2578), ACE inhibitor GPAGAPGAA (ID 3363), and antibacterial peptide ALCSEK (ID 4011). These peptides have no common fragments (subsequences). The use of incomplete InChIKey with the exact search option will fail to produce any results. 

The sequence-based search is the most common and most intuitive option used to find peptide information in the database [4]. The BIOPEP-UWM database offers an opportunity to find all longer sequences containing a query fragment and to find a given sequence (exact). The first opportunity allows user to find peptides containing a defined continuous motif, e.g., attributed to the given function [74,75]. This search option also follows the fragmentomics concept [76]. It assumes that shorter (functional) bioactive subsequences present in a sequence may be crucial for the biological activity of the entire peptide molecule (peptide). Examples of peptides inscribing into this concept may be found in the BIOPEP-UWM (e.g., hemorphins or ACE inhibitors from caseins) and in other peptide databases such as EROP-Moscow [20], PepBank [67], SATPdb [70] or AHTPDB [45]. The exact search option is sufficient to check the bioactivity of peptides identified among protein hydrolysis products. An example of such an experiment has recently been described by Martini et al. [77] and Garcia-Vaquero et al. [78].

In the case of the property-based search (involving the number of amino acid residues or molecular mass range), choosing the exact search option does not change the output. We generally recommend using the exact search option for the sequence-based search.

## 6. Analysis

The “analysis” page includes the following tabs: “profiles of potential biological activity”, “calculations”, “enzyme(s) action”, “find”, “batch processing”, “definitions”, “SMILES”, and “find the enzyme for peptide release” (Supplementary Figure S6).

The profile of a potential biological activity is defined as the type and location of bioactive fragments in a protein or a peptide chain [79]. This idea is based on the assumption that the same bioactive fragment, especially a short one (2–3 amino acid residues), cannot be attributed to a given protein, but may be present in many sequences (many form the so-called common subsequences) [75,79]. The concept of profiles of the potential activity of peptide fragments is consistent with the fragmentomic approach proposed by Zamyatnin [76] (see above). The profiles of potential biological activity of proteins can be obtained using the asterisk by default. Examples of published profiles of the potential activity of peptide or protein fragments may be found in publications of Bauchart et al. [80], Huang et al. [81], Tapal et al. [82], Khazaei et al. [17], and Jakubczyk et al. [83]. The profile may also be constructed for the specific bioactivity (bioactivity of interest) when selecting the activity instead of an asterisk from a toolbar. The menu to be used for the construction of potential biological activity profiles is shown in Supplementary Figures S7–S9. The profile of a potential biological activity of a protein or a peptide sequence is presented as a table including the following columns: ID, name of peptide, activity, number of repetitions of a particular bioactive fragment in a query sequence, sequence of the bioactive fragment, and location of the bioactive fragment in a query sequence. An example of the above profile is presented in Supplementary Table S2.

The “calculations” tab enables calculating two quantitative parameters that characterize proteins as potential precursors of bioactive peptides: the frequency of bioactive fragments occurrence in a protein sequence (A) and a potential biological activity of protein fragments (B). Equations 1 and 2 enabling calculation of the above parameters are provided in Table 5. The menu of the “calculations” tab is shown in Supplementary Figure S10. An example of the output is presented in Supplementary Table S3. The frequency of bioactive fragments occurrence in a protein sequence (A) is calculated for all bioactive peptides present in the query sequence (using the asterisk) or for one specific peptide (by choosing the bioactivity from a toolbar). Potential biological activity of protein fragments (B) may be calculated only if peptide IC_50_ or EC_50_ is available. The program skips peptides without known IC_50_ or EC_50_ value. For instance, Supplementary Table S3 provides B values for ACE and DPPIV inhibitors only. In the case of other activities, B values have not been calculated due to the lack of IC_50_ or EC_50_ attributed to particular peptides. Articles published by Udenigwe et al. [84] and Lin et al. [85] contain representative results of calculations of quantitative parameters characterizing food proteins as potential precursors of bioactive peptides.

**Figure 2:**
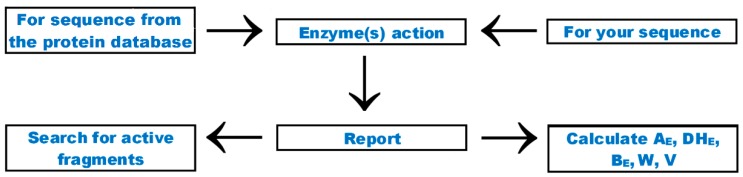
Scheme of the “enzyme(s) action” tab. Option (see Figure 1) “search for enzyme with given specificity” is not included in the Figure. A screenshot of the menu of this tab is presented in Supplementary Figure S11.

The “Enzyme(s) action” tab allows simulating proteolysis catalyzed by endopeptidases. The scheme of steps required to obtain the peptides potentially released by a given enzyme (or enzymes) is presented in Figure 2. Screenshots of menus of particular tabs are presented in Supplementary Figures S11–S16. The menu also enables enzyme choice (Supplementary Figures S12 and S13). It allows the simulation of proteolysis using one to three enzymes. Example information about a single enzyme (plasmin; EC 3.4.21.7; MEROPS ID: S01.233) is presented in Supplementary Figure S14. The enzyme is annotated using a connection ID, indicating a single peptide bond hydrolyzed by the enzyme and enzyme ID. One enzyme may cover few connection IDs (in the case of plasmin—two). Enzyme specificity is described using two terms: a recognition sequence understood as a fragment of an amino acid sequence recognized by the proteolytic enzyme and a cutting sequence understood as an amino acid residue preceding or following the bond hydrolyzed by protease [8]. The recognition sequence may contain a single amino acid residue (e.g., for plasmin) or a longer fragment such as for a ginger protease—zingipain (EC 3.4.22.67; MEROPS ID: C01.017). Annotations “C-terminus” and “N-terminus” indicate bonds formed by a carboxyl and amine group of an amino acid residue, respectively, hydrolyzed by the enzyme. Data concerning particular enzymes contain references: databases such as MEROPS [41] and CutDB [56] or publications (Bastian and Brown [89] for plasmin and Huang et al. [90] for zingipain). Apart from the addition of new enzymes, the specificity has recently been modified for some of the existing ones. The modification included the addition of new recognition sequences and cutting sequences (possessing connection IDs within the range 141–184), according to data presented in the so-called specificity matrices in the MEROPS database. These matrices are continuously updated to follow newly appearing information about new sites susceptible to proteolysis in protein sequences [41]. Proteolysis simulation is simplified. It assumes that all bonds theoretically susceptible to a given proteinase are hydrolyzed. In real experiments, the proteolysis is often incomplete. This finding may explain false-positive results, i.e., lack of expected peptides. False-negative results may be explained by incomplete knowledge about proteolytic specificity, i.e., the situation when some bonds susceptible to the proteolytic enzyme are considered resistant. The addition of new recognition and cutting sequences to the enzyme data aims to minimize the occurrence of false-negative results. 

Results of simulated proteolysis of an example peptide can be found in Supplementary Figure S15. Displayed results of the initial step of simulation include sequences of peptides being products of proteolysis and their location in the precursor sequence. The next step may include the search for bioactive peptides among products of simulated proteolysis or calculation of quantitative parameters characterizing the proteolysis (Figure 2 and Figure S15). The parameters available via the “enzyme(s) action” tab are calculated according to Equations (3)–(7) from Table 5. Representative results of the search for active peptides among simulated proteolysis products and calculation of quantitative parameters are presented in the Supplement (Tables S4 and S5, respectively). Calculation of parameters B_E_ and V involves EC_50_ or IC_50_ values. If they are not available, peptides are not taken into account. Simulation of proteolysis using the BIOPEP-UWM database has recently been described by, e.g., Lin et al. [85], Yu D. et al. [91], and Kandemir-Cavas et al. [92]. Data concerning proteolysis simulation may be interpreted together with protein structures [93].

A new tab named “search for enzymes with given specificity” enables the search for information about an enzyme using recognition sequence, cutting sequence, and choice between C- and N-terminus (bond formed by carboxyl or amine group of amino acid residue, respectively). Results include the list of enzymes with a given specificity. An example of a query and result produced using the above option may be found in Supplementary Figure S16 and Supplementary Table S6. For most of the enzymes, the recognition sequence contains only one amino acid residue. 

The content of the new “find” tab enables quickly finding of some information in protein and bioactive peptide databases. Particular tabs enable display of a full list of protein sequences annotated in the BIOPEP-UWM database, a full list of peptides revealing a given activity, and a list of all proteins or peptides containing the query sequence (see Supplementary Figure S17). The last option enables finding all proteins or peptides containing a given bioactive fragment or a recognition sequence available for the proteolytic enzyme. An example result of a search for a VPP sequence in the database of bioactive peptides is presented in Table S7 (Supplement). Results cover links to peptide or protein data, ID number, name, and sequence.

**Figure 3:**
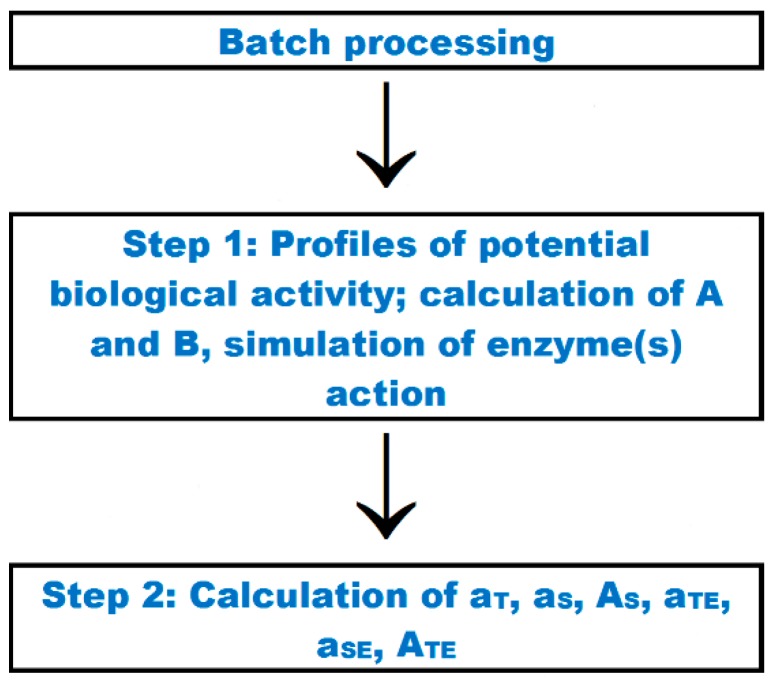
Scheme of the “batch processing” tab action.

Another new “batch processing” option serves for the simultaneous processing of a set of few sequences of proteins or peptides being potential precursors of bioactive peptides. The total length of all sequences forming the query set may be up to c.a. 1500 amino acid residues. The scheme of activities available via this option is presented in Figure 3. The screenshot of the input window is available in Supplementary Figure S18. The FASTA format [94] is used to input a set of sequences. The “batch processing” option enables performing any action available via the tabs: “profiles of potential biological activity”, “calculations”, and “enzyme(s) action”. Moreover, there are new parameters characterizing the occurrence and possibility of enzymatic release of an individual peptide from few precursor sequences (a_T_, a_S_, A_S_, a_TE_, a_SE_, A_TE_) calculated according to Equations (8)–(10) and (12)–(14) in Table 5. Distribution of particular fragments in the set of sequences may be in the focus of scientific interests when using in silico methodologies [76,77,95]. Analysis may cover all possible or selected options. Supplementary Figure S18 shows a set of sequences ready for an analysis concerning bioactive peptides (excluding options concerning data from the database of allergenic proteins). The batch analysis is performed in two steps (Figure 3). The first step may be performed for all activities (default option) or a selected one. The second step may be performed after the first one had been completed. The parameters may be calculated for all bioactive fragments found in the set of sequences or for manually selected peptides. Results of the first and the second step of batch analysis are presented in Supplementary Tables S8 and S9, respectively.

The “definitions” tab summarizes terms and definitions used in the BIOPEP-UWM database including equations used to calculate quantitative parameters, as shown in Table 5.

The “SMILES” tab, introduced in 2018, enables translating amino acid sequences (written using standard one-letter code) into the chemical language “SMILES”. SMILES representations are built according to a simplified algorithm described by Siani et al. [96]. SMILES codes of particular amino acid residues are written using the same layout as used in the SwissSidechain database [72] and source codes of the CycloPs program [97] (program temporarily unavailable). The procedure was tested and verified according to recommendations proposed in our previous publication [98]. The MarvinSketch 17.28 software (ChemAxon, Budapest, Hungary) was used to test and verify SMILES strings of peptides. The application utilizes the sequences of peptides built from 20 protein amino acids, their D-enantiomers, L- and D-phosphoserine (Symbols B and b, respectively), and C-terminal amide group. It is easy and fast in use and can process linear peptides only. Disulfide bonds and other modifications may be inserted using molecule editors (e.g., MarvinSketch, Dendrimer Builder program provided by the University of Bern, Switzerland, and molecule editor of the NANPDB [65] database) which may serve as alternatives to our application. The first one may be used to construct any molecules from building blocks drawn or imported as SMILES strings, the second, to build representations of branched peptides containing some non-protein amino acids, whereas the third to encode pyrrolysine and selenocysteine apart from 20 most common protein amino acids. Our application converts amino acid sequences into the so-called aromatic SMILES. Some search engines do not utilize this version [30]. The aromatic version of the SMILES string may be converted into an alternative, so-called Kekule version using, e.g., the molecule editor of the PubChem database [99] or MarvinSketch software. Screenshots of the “SMILES” tab window with query and result are given in the Supplementary Figures S19 and S20, respectively. Two types of SMILES representations of the example peptide may be found in Supplementary Table S10.

**Figure 4:**
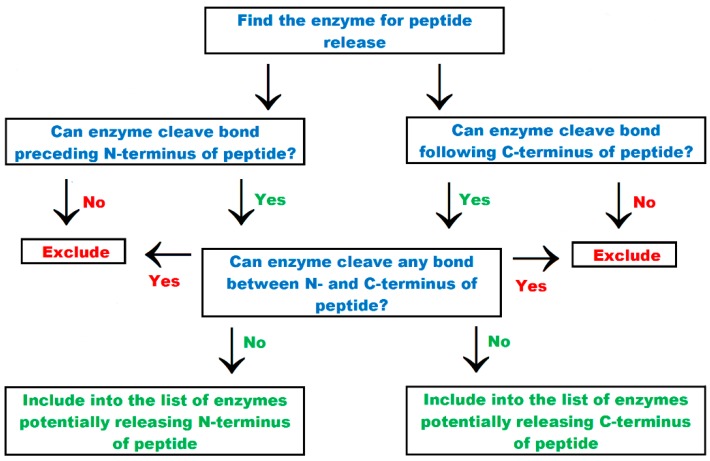
Scheme of “find enzyme for peptide release” tab action.

The way of understanding the output information when using the new tab entitled “find the enzyme for peptide release” is summarized in Figure 4. The screenshot of the menu of this tab and representative results are shown in the Supplementary Figure S21 and Supplementary Table S11, respectively. The input includes peptide sequences provided in FASTA format and the precursor (protein or peptide) sequence. The output includes a list of all enzymes with the specificity sufficient to catalyze particular proteolytic events. A proteolytic event is understood as a case of cleavage of an individual peptide bond. This term has been introduced in the CutDB database [56]. Release of a peptide from the precursor sequence requires two proteolytic events: cleavage of bond preceding N- and following C-terminus (indicated in Supplementary Table S11 as N and C, respectively). If a given peptide appears in the precursor sequence more than once, then the particular events attributed to this peptide are indicated as 1N, 1C, 2N, 2C, and so forth. An example peptide with the AP sequence occurs in the precursor sequence RWAFAPGFAPGHIP twice (positions 5–6, 9–10). Its release is associated with four proteolytic events: 1N—cleavage of the bond between the residues 4 and 5, 1C—cleavage of the bond between the residues 6 and 7, 2N—cleavage of the bond between the residues 8 and 9, and 2C—cleavage of the bond between the residues 10 and 11. Displayed results concerning enzyme catalyzing the particular proteolytic events cover the following data: name, EC number, enzyme ID in the BIOPEP-UWM database, connection ID, cutting sequence, and recognition sequence. The cutting sequences are described using the symbols “+” and “−“ assigned to the amino acid symbols. Symbol “+” means that the amino acid residue follows the cleaved bond, i.e., this bond is formed by the amine group. For example, the symbol “A+” means that the cleaved bond is formed by the amine group of alanine. The symbol “−“ means that the amino acid residue is located before the cleaved bond, i.e., this bond is formed by the carboxyl group of the amino acid. For instance, the symbol “W-“ means a bond formed by the carboxyl group of tryptophan. Enzymes releasing N- and C-terminus are summarized separately. This solution may be justified by the fact that peptides may be released by more than one enzyme (N- and C- terminus are not released by the same enzyme). This process can be exemplified by protein digestion in the human gastrointestinal tract [100].

## 7. Useful Links and Other Tabs

The BIOPEP-UWM plays the role of a metaserver enabling access to databases and software useful in research concerning peptides and proteins. The linked tools available via the “useful links” tab (Figure 1; Supplementary Figure S1) are divided into categories according to Minkiewicz et al. [101]. These categories are summarized in Table 6.

Other tabs available from the BIOPEP-UWM main page are as follows: List of publications of our group concerning the BIOPEP-UWM database, brief summary concerning the database (“about BIOPEP-UWM” tab), publications concerning particular parts of the BIOPEP-UWM database recommended to be cited by users, and contact data of database curators.

## 8. Final Remarks

This paper presents the current status of the BIOPEP-UWM^TM^ database including changes introduced within the period of 2016–2019. Apart from the addition of new peptides (562 items added since submission of our last publication describing the database of sensory peptides and amino acids [9]), information about the existing ones has been completed (especially chemical codes and database references). We also added several new options that are summarized in the Table 7. 

The content of this publication is not restricted to description of new changes in the database and associated tools during the last three years. We try to provide a complete description including both old and new options.

The next modifications would be aimed at removing the weak points of the database and associated applications. We would like to ask users to submit new peptides (via the current version of the “submit new peptide” tab) and any remarks helpful in improving the bioinformatic tool described in this paper.

## Figures and Tables

**Table 1:** Content of a page of a representative peptide.

**ID**	9473		
**Name**	ACE inhibitor		
**Sequence**	GHS		
**InChIKey**	LPCKHUXOGVNZRS-YUMQZZPRSA-N		
**Function**	Inhibitor of Angiotensin-Converting Enzyme (ACE) (EC 3.4.15.1) (MEROPS ID: M02-001)		
**Number of Amino Acid Residues**	3	**Activity Code**	ah
**Activity**	ACE inhibitor		
**Chemical Mass**	299.2740	**Monoisotopic Mass**	299.1110
**IC_50_**	0.00 µM		
**Bibliographic Data**			
**Authors**	He R., Malomo S. A., Alashi A., Girgih A. T., Ju X., Aluko R. E.		
**Title**	Glycinyl-histidinyl-serine (GHS), a novel rapeseed protein-derived peptide, has a blood pressure-lowering effect in spontaneously hypertensive rats. J. Agric. Food Chem., 61, 8396-8402, 2013		
**Year**	2013	**Source**	Journal
**Additional Information**			
BIOPEP-UWM database of bioactive peptides SMILES: NCC(=O)N[C@@H](Cc1c[nH]cn1)C(=O)N[C@@]([H])(CO)C(=O)O InChI=1S/C11H17N5O5/c12-2-9(18)15-7(1-6-3-13-5-14-6)10(19)16-8(4-17)11(20)21/h3,5,7-8,17H,1-2,4,12H2,(H,13,14)(H,15,18)(H,16,19)(H,20,21)/t7-,8-/m0/s1 InChIKey: LPCKHUXOGVNZRS-YUMQZZPRSA-N Inhibitor of Renin (EC 3.4.23.15) (MEROPS ID: A01.007) according to the BIOPEP-UWM database of bioactive peptides (ID 9472)			
**Database Reference**			
AHTPDB: ID 1053, 2949 BioPepDB: ID biopep00354 BIOPEP-UWM database of bioactive peptides: ID 9472 SATPdb: ID satpdb13065			

**Table 2:** List of activities of peptides annotated in the BIOPEP-UWM database of bioactive peptides.

Activity	Description ^1^
ACE inhibitor ^2^	Inhibitors of angiotensin-converting enzyme (ACE) (EC 3.4.15.1) (MEROPS ID: M02-001)
activating ubiquitin-mediated proteolysis	Peptides activating proteolysis mediated by ubiquitin
alpha-amylase inhibitor ^2^	Inhibitors of α-amylase (EC 3.2.1.1)
alpha-glucosidase inhibitor ^2^	Inhibitors of α-glucosidase (EC 3.2.1.20)
anorectic	Peptides causing a decrease in food intake and suppression of appetite.
antiamnestic	Inhibitors of prolyl oligopeptidase (EC 3.4.21.26) (MEROPS ID: S09.001). The enzyme catalyzes degradation of neuropeptides, e.g., involved in processes associated with memory.
antibacterial	Peptides revealing any action against bacteria
anticancer	Peptides revealing any action against cancers
antifungal	Peptides revealing any action against fungi
anti-inflammatory	Peptides reducing inflammation or swelling
antioxidative	Peptides inhibiting oxidation
antithrombotic	Inhibitors of blood coagulation. Inhibitors of thrombin (EC 3.4.21.5) (MEROPS ID: S01.217) are attributed to this activity.
antiviral	Peptides revealing any action against viruses. Inhibitors of viral enzymes are included.
bacterial permease ligand	Ligands of bacterial permeases
binding ^2^	Peptides binding any biomolecules. Mineral binding peptides are also attributed to this activity.
CaMKII inhibitor ^2^	Inhibitors of Ca^2+^/calmodulin-dependent protein kinase (CaMKII) (EC 2.7.11.17)
CaMPDE inhibitor ^2^	Inhibitors of 3′,5′-cyclic-nucleotide phosphodiesterase (Calmodulin-dependent phosphodiesterase 1—CaMPDE) (EC 3.1.4.17)
chemotactic	Peptides inducing chemotaxis, i.e. movement in response to a chemical stimulus
celiac toxic	Peptides toxic to people suffering from celiac disease
contracting	Peptides stimulating muscle contraction
dipeptidyl peptidase III inhibitor ^2^	Inhibitors of dipeptidyl peptidase III (EC 3.4.14.4) (MEROPS ID M49.001)
dipeptidyl peptidase IV inhibitor ^2^	Inhibitors of dipeptidyl peptidase IV (EC 3.4.14.5) (MEROPS ID S09.003)
embryotoxic	Peptides toxic to animal embryos
hemolytic	Peptides destroying red blood cells
heparin binding ^2^	Heparin binding peptides
HMG-CoA reductase inhibitor ^2^	Inhibitors of 3-hydroxy-3-methyl-glutaryl-coenzyme A reductase (HMG-CoA reductase) (EC 1.1.1.34)
hypotensive	Peptides causing blood pressure decrease
immunomodulating	Peptides modulating activity of the immune system
immunostimulating	Peptides stimulating activity of the immune system
inhibitor ^2^	Peptides inhibiting various biological processes. Information about processes is provided on the pages of individual peptides.
membrane-active ^2^	Peptides affecting transmembrane transport
natriuretic	Peptides inducing the excretion of sodium by kidneys (natriuresis)
neuropeptide	Peptides affecting activity of the nervous system
opioid	Ligands of opioid receptors
opioid agonist	Agonists of opioid receptors
opioid antagonist	Antagonists of opioid receptors
orphan receptor GPR14 agonist	Agonists of orphan receptor GPR14
Protein Kinase C inhibitor ^2^	Inhibitors of protein kinase C (EC 2.7.11.13)
regulating	Peptides regulating various biological processes. Information about processes is provided on the pages of individual peptides.
renin inhibitor ^2^	Inhibitors of renin (EC 3.4.23.15) (MEROPS ID A01.007)
stimulating	Peptides stimulating various biological processes. Information about processes is provided on the pages of individual peptides.
toxic ^2^	Toxic peptides
vasoconstrictor	Peptides causing blood pressure increase

^1^ More information concerning enzymes inhibited by peptides is available in the following databases: ExplorEnz [38], BRENDA [39], ChEMBL [40], and MEROPS [41]. Information about associations between abnormal enzyme activity and diseases may be found in the OpenTargets database [42]. ^2^ Activities absent in the version described in our publication from 2008 [8].

**Table 3:** Databases cited on the “Database reference” page and other bioinformatic tools mentioned in the publication.

Database Name	Website ^1^	Reference
ACToR ^2^	https://actor.epa.gov/actor/home.xhtml	[43]
AHTPDB ^2^	https://webs.iiitd.edu.in/raghava/ahtpdb/	[45]
APD	http://aps.unmc.edu/AP/main.html	[46]
BindingDB ^2^	http://www.bindingdb.org/bind/index.jsp	[47]
BioPepDB	http://bis.zju.edu.cn/biopepdbr/	[48]
BitterDB ^2^	http://bitterdb.agri.huji.ac.il/dbbitter.php	[49]
Brainpeps	http://brainpeps.ugent.be/	[50]
BRENDA ^1^	https://www.brenda-enzymes.org/	[39]
CAMP	http://www.camp.bicnirrh.res.in/	[51]
CancerPPD	http://crdd.osdd.net/raghava/cancerppd/index.php	[52]
ChEBI ^2^	https://www.ebi.ac.uk/chebi/	[53]
ChEMBL ^2^	https://www.ebi.ac.uk/chembl/	[40]
ChemIDplus ^2^	https://chem.nlm.nih.gov/chemidplus/chemidlite.jsp	[44]
ChemSpider ^2^	http://www.chemspider.com/Default.aspx	[54]
CompTox	https://comptox.epa.gov/dashboard	[55]
CutDB	http://cutdb.burnham.org	[56]
DBAASP	https://dbaasp.org/	[57]
Dendrimer Builder	http://dendrimerbuilder.gdb.tools/	*
DrugBank ^2^	https://www.drugbank.ca/	[58]
EROP-Moscow ^2^	http://erop.inbi.ras.ru/	[20]
ExplorEnz	https://www.enzyme-database.org/index.php	[38]
FeptideDB	http://www4g.biotec.or.th/FeptideDB/index.php	[13]
FooDB ^2^	http://foodb.ca/	*
Hemolytik	http://crdd.osdd.net/raghava/hemolytik/	[59]
HMDB ^2^	http://www.hmdb.ca/	[60]
J-Global	https://jglobal.jst.go.jp/en/	*
KEGG ^2^	https://www.genome.jp/kegg/	[61]
LabWorm	https://labworm.com/	*
MarvinSketch	https://chemaxon.com/products	*
MBPDB	http://mbpdb.nws.oregonstate.edu/	[62]
MEROPS ^2^	https://www.ebi.ac.uk/merops/	[41]
MetaboLights	https://www.ebi.ac.uk/metabolights/index	[63]
MetaComBio ^2^	http://www.uwm.edu.pl/metachemibio/index.php/about-metacombio	[11]
MilkAMP	http://milkampdb.org/home.php	[64]
NANPDB	http://african-compounds.org/nanpdb/	[65]
NeuroPep	http://isyslab.info/NeuroPep/	[66]
omicX	https://omictools.com/	*
OpenBabel ^2^	http://openbabel.org/wiki/Main_Page	[32]
OpenTargets	https://www.targetvalidation.org/	[42]
PepBank ^2^	http://pepbank.mgh.harvard.edu/	[67]
PeptideDB	http://www.peptides.be/	[68]
ProPepper	https://propepper.net/	[69]
PubChem ^2^	https://pubchem.ncbi.nlm.nih.gov/	[22]
SATPdb ^2^	http://crdd.osdd.net/raghava/satpdb/links.php	[70]
SpirPep	http://spirpepapp.sbi.kmutt.ac.th/SpirPep/Home	[12]
SureChEMBL	https://www.surechembl.org/search/	[71]
SwissSidechain	https://swisssidechain.ch/	[72]
ZINC ^2^	http://zinc.docking.org/	[73]

^1^ Accessed in July and August 2019. ^2^ Tools cited in our previous publication [9]. * No reference available.

**Table 4:** List of search options available in the BIOPEP-UWM database of bioactive peptides. Options described in this table have been announced in [30].

Search Option	Output	
	Version without Exact Search	Version with Exact Search ^1^
ID	Peptide with given ID	
Name	List of all peptides with the name containing the given word (words)	Peptide with the given name (may appear more than once if it is annotated with more activities)
Activity	Complete list of peptides with all activities named using the given word (e.g., inhibitor)	List of all peptides with the given activity
Mass	List of all peptides having molecular masses within the given range (e.g., 500–600)	
Reference	List of all peptides described in articles published by the given author (or authors with the same second name)	
Sequence	List of all peptides with sequences containing the given fragment	Peptide with the given sequence (may appear more than once if it is annotated with more activities). ^2^
Number of amino acid residues	List of all peptides containing the given number of amino acid residues (e.g., 3)	
InChIKey ^1^	Peptide with the given InChIKey. Peptide exhibiting more than one activity annotated in the BIOPEP-UWM will appear more than once ^2^	

^1^ New search options. ^2^ These options give equivalent search results.

**Table 5:** Quantitative parameters characterizing proteins as potential precursors of bioactive peptides, available in the BIOPEP-UWM database.

Equation No.	Parameter	Reference
1. ^1^	The frequency of bioactive fragments occurrence in a protein sequence (A) A = a/N a—the number of fragments with a given activity, N—the number of amino acid residues	[86]
2. ^1^	Potential biological activity of protein fragments (B) [μM^−1^] B = [Σ(a_i_/EC_50i_)]/N or B = [Σ(a_i_/IC_50i_)]/N a_i_—the number of repetitions of i-th bioactive fragment in a protein sequence, EC_50i_—the concentration of i-th bioactive peptide corresponding to its half-maximal activity [µM], IC_50i_—the concentration of i-th bioactive peptide corresponding to half-maximal inhibition [µM], N—the number of amino acid residues	[86]
3. ^2^	The frequency of release of fragments with a given activity by selected enzymes (A_E_) A_E_ = d/N d—the number of peptides with a given activity (e.g., ACE inhibitors) released by a given enzyme (e.g., trypsin) N—the number of amino acid residues in protein	[87]
4. ^2^	The relative frequency of release of fragments with a given activity by selected enzymes (W) W = A_E_/A A_E_—the frequency of release of fragments with a given activity by selected enzymes (from Equation (3)) A—the frequency of bioactive fragments occurrence in a protein sequence (from Equation (1))	[87]
5. ^2^	Activity of fragments potentially released by proteolytic enzyme (enzymes) (B_E_) B_E_ = [Σ(d_j_/EC_50j_)]/N or B_E_ = [Σ(d_j_/IC_50j_)]/N d_j_—the number of repetitions of j-th bioactive fragment released by a given enzyme (enzymes) from a protein sequence, EC_50j_—the concentration of j-th bioactive peptide corresponding to its half-maximal activity [µM], IC_50j_—the concentration of j-th bioactive peptide corresponding to half-maximal inhibition [µM], N—the number of amino acid residues in a protein chain	*
6. ^2^	Relative activity of fragments potentially released by proteolytic enzyme (enzymes) (V) V = B_E_/B B_E_—activity of fragments potentially released by proteolytic enzyme (enzymes) (from Equation (5)) B—potential biological activity of protein fragments (from Equation (2))	*
7. ^2^	Theoretical degree of hydrolysis (DH_T_) DH_T_ = d/D × 100% d—number of hydrolyzed peptide bonds in a protein/peptide chain D—total number of peptide bonds in a protein/peptide chain	[88]
8. ^3^	The number of repetitions of the bioactive fragment in all sequences of the protein/peptide set analyzed (a_T_) a_T_ = a_1_ + a_2_ + … + a_L_ a_1_—a_L_—the number of repetitions of a given bioactive fragment in particular sequences in the dataset submitted for analysis L—the number of sequences in the protein/peptide set analyzed	*
9. ^3^	The number of repetitions of a given fragment in all sequences of the selected protein/peptide fraction (a_S_) a_S_ = a_T_/L a_T_—the number of repetitions of the bioactive fragment in all sequences of the protein/peptide set analyzed (from Equation (8)) L—the number of sequences in the protein/peptide set analyzed	*
10. ^3^	The mean frequency of the occurrence of a single fragment in a sequence of protein/peptide classified to a given group (A_S_) A_S_ = a_T_/N_T_ a_T_—the number of repetitions of the bioactive fragment in all sequences of the protein/peptide set analyzed N_T_—the total number of amino acid residues in all protein/peptide sequences belonging to the set (from Equation 10)	*
11. ^4^	The total number of amino acid residues in all protein/peptide sequences belonging to the set (N_T_) N_T_ = N_1_ + N_2_ + … + N_L_ N—the number of amino acid residues in a single protein/peptide chain L—the number of protein/peptide chains in the set	*
12. ^3^	The number of cases of release of the bioactive fragment from all sequences of the protein/peptide set analyzed (a_TE_) a_TE_ = a_1E_ + a_2E_ + … + a_LE_ a_1E_—a_LE_—the number of cases of release of the bioactive fragment from particular sequences of the protein/peptide set analyzed L—the number of protein/peptide chains in the set	*
13. ^3^	Mean number of cases of predicted release of a single fragment by a selected enzyme from the chain of protein/peptide belonging to the set analyzed (a_SE_) a_SE_ = a_TE_/L a_TE_—the number of cases of release of the bioactive fragment from all sequences of the protein/peptide set analyzed L—the number of protein/peptide chains in the set	*
14. ^3^	Predicted frequency of release of a single peptide by proteolytic enzyme from the set of protein/peptide sequences analyzed (A_SE_) A_TE_ = a_TE_/N_T_ a_T_—the number of cases of release of the bioactive fragment from all sequences of the protein/peptide set analyzed N_T_—the total number of amino acid residues in all protein/peptide sequences belonging to the set (from Equation (10))	*

^1^ available via the “profiles” tab and “batch processing” tab. ^2^ available via the “enzyme (s) action” tab and “Batch processing” tab. ^3^ available via the “batch processing” tab only. ^4^ not displayed among the results. Shown only to explain the calculation of other parameters. * New parameters described for the first time in this publication. Some of them have been announced in [4].

**Table 6:** Categories of bioinformatic tools available via the “useful links“ tab.

Category	Description
Bioactive peptide databases	Databases of biologically active peptides including general databases (covering several activities) or databases of particular activities (e.g., antimicrobial)
Bioactivity prediction	Software predicting biological activity of peptides, especially interactions with proteins, e.g., enzymes
Immunology of proteins and peptides	Databases of allergens and epitopes, software for predicting allergenicity and occurrence of epitopes as well as other software from the area of immunology
Literature data mining	Software supporting search for biomedical data (e.g., concerning proteins and peptides) in literature
Miscellaneous	Databases and software not belonging to other categories. Chemical databases and metabases are attributed to this category.
Motifs	Programs enabling constructing sequence motifs and finding them in protein or peptide sequences
Physicochemical properties	Software used to predict and exploit the physicochemical properties of peptides
Prediction of post-translational modifications	Software used to predict the location of post-translational modifications (phosphorylation, glycosylation) in protein and peptide sequences
Programs supporting peptide design	Software supporting design of peptides with desired biological properties
Protein resources	Databases and software concerning proteins but not peptides, including databases of protein sequences and structures
Proteolysis	Databases annotating proteolytic enzymes, software for proteolysis simulation
Proteomic tools	Tools supporting proteomics research including mass spectrometry
Sequence alignments	Software for constructing protein and peptide sequence alignments and for searching in protein sequence databases
Structure prediction and visualization	Software for modeling secondary and tertiary structures of proteins and peptides

**Table 7:** New options in the BIOPEP-UWM database and modifications of existing ones, not described in the previous publications [8,9].

Option	Description
Peptide annotation	Possibility of annotation of peptides containing D-amino acids
Search options ^1^	Search on the basis of InChIKey; addition of “exact match” search as user’s choice, designed especially for sequence search
List of peptide activities	List of peptide activities rearranged and enriched
Proteolytic enzyme annotation	Updated list of bonds susceptible to proteolytic enzyme action
New search options	Search on the basis of InChIKey; addition of “exact match” search as user’s choice
“SMILES” tab ^1^	Application converting amino acid sequences into the SMILES code
New options available via the “enzyme(s) action” tab	New quantitative parameters describing possibility of release of bioactive peptides by proteolytic enzymes—Equations (5)–(7) in Table 5, option enabling finding enzyme with a given specificity among proteinases annotated in the database
“find the enzymes for peptide release” tab	Option which enables finding proteolytic enzymes liberating of N- and C-termini of bioactive peptides
“find” tab	Shortcut to the list of peptides with a given activity
Batch processing	Option which enables finding profiles of potential biological activity of fragments, calculating quantitative parameters that characterize protein or peptide, and simulating proteolysis for a set of sequences
Quantitative parameters characterizing occurrence and possibility of release of bioactive peptide from a set of sequences	Parameters calculated via the “batch processing” option—Equations (8)–(10) and (12)–(14) in the Table 5
The “BIOPEP-UWM news” tab	Tab designed to provide important news concerning the database

^1^ Application serving for conversion amino acid sequences into SMILES code has been announced in [4].

## Supplementary Materials

Supplementary materials can be found at https://www.mdpi.com/1422-0067/20/23/5978/s1.

## Abbreviations

ACE	Angiotensin-converting enzyme (EC 3.4.15.1)
ACToR	Aggregated Computational Toxicology Online Resource
AHTPDB	Antihypertensive Peptide Database
APD	Antimicrobial peptide database
BioPepDB	Bioactive Peptide Database
BRENDA	Braunschweig Enzyme Database
CAMKII	Ca^2+^/calmodulin-dependent protein kinase (EC 2.7.11.17)
CAMP	Collection of antimicrobial peptides
CaMPDE	Calmodulin-dependent phosphodiesterase 1 (EC 3.1.4.17)
CancerPPD	Anticancer protein and peptide database
CAS	Chemical Abstract Service provided by American Chemical Society
CID	Compound Identifier (in PubChem database)
DB	database
DBAASP	Database of Antimicrobial Activity and Structure of Peptides
EBI	European Bioinformatics Institute
EC_50_	Concentration corresponding to half-maximal activity
EMBL	European Molecular Biology Laboratory
EROP	Endogenous Regulatory Oligopeptide knowledgebase
FeptideDB	Food Peptide Database
GPR14	Abbreviation of urotensin II receptor
HMDB	Human Metabolome Database
IC_50_	Concentration corresponding to half-maximal inhibition
InChI	International Chemical Identifier
HMG-CoA	3-hydroxy-3-methyl-glutaryl-coenzyme A (PubChem CID: 445127; CAS registry No 1553-55-5)
InChIKey	Key of International Chemical Identifier
IUPAC	International Union of Pure and Applied Chemistry
KEGG	Kyoto Encyclopedia of Genes and Genomes
MBPDB	Milk Bioactive Peptide Database
MetaComBio	Meta Compound Bioactivity
MilkAMP	Milk antimicrobial peptide database
SATPdb	Structurally Annotated Therapeutic Peptide database
SMILES	Simplified Molecular Input Line Entry System or Simplified Molecular Input Line Entry Specification
UWM	University of Warmia and Mazury

## References

[1] Holton T.A., Vijayakumar V., Khaldi N. (2013). Bioinformatics: Current perspectives and future directions for food and nutritional research facilitated by a food-wiki database. Trends Food Sci. Technol..

[2] Udenigwe C.C. (2014). Bioinformatics approaches, prospects and challenges of food bioactive peptide research. Trends Food Sci. Technol..

[3] Iwaniak A., Minkiewicz P., Darewicz M., Protasiewicz M., Mogut D. (2015). Chemometrics and cheminformatics in the analysis of biologically active peptides from food sources. J. Funct. Foods.

[4] Iwaniak A., Darewicz M., Mogut D., Minkiewicz P. (2019). Elucidation of the role of in silico methodologies in approaches to studying bioactive peptides derived from foods. J. Funct. Foods.

[5] Agyei D., Tsopmo A., Udenigwe C.C. (2018). Bioinformatic and peptidomic approaches to the discovery and analysis of food-derived bioactive peptides. Anal. Bioanal. Chem..

[6] Kalmykova S.D., Arapidi G.P., Urban A.S., Osetrova M.S., Gordeeva V.D., Ivanov V.T., Govorun V.M. (2018). In silico analysis of peptide potential biological functions. Russ. J. Bioorg. Chem..

[7] Tu M., Cheng S., Lu W., Du M. (2018). Advancement and prospects of bioinformatics analysis for studying bioactive peptides from food-derived protein: Sequence, structure, and functions. Trends Anal. Chem..

[8] Minkiewicz P., Dziuba J., Iwaniak A., Dziuba M., Darewicz M. (2008). BIOPEP database and other programs for processing bioactive peptide sequences. J. AOAC Int..

[9] Iwaniak A., Minkiewicz P., Darewicz M., Sieniawski K., Starowicz P. (2016). BIOPEP database of sensory peptides and amino acids. Food Res. Int..

[10] Piovesana S., Capriotti A.L., Cavaliere C., La Barbera G., Montone C.M., Chiozzi R.Z., Laganà A. (2018). Recent trends and analytical challenges in plant bioactive peptide separation, identification and validation. Anal. Bioanal. Chem..

[11] Minkiewicz P., Iwaniak A., Darewicz M. (2015). Using internet databases for food science organic chemistry students to discover chemical compound information. J. Chem. Educ..

[12] Anekthanakul K., Hongsthong A., Senachak J., Ruengjitchatchawalya M. (2018). SpirPep: An in silico digestion-based platform to assist bioactive peptides discovery from a genome-wide database. BMC Bioinform..

[13] Panyayai T., Ngamphiw C., Tongsima S., Mhuantong W., Limsripraphan W., Choowongkomon K., Sawatdichaikul O. (2019). FeptideDB: A web application for new bioactive peptides from food protein. Heliyon.

[14] Dziuba M., Minkiewicz P., Dąbek M. (2013). Peptides, specific proteolysis products as molecular markers of allergenic proteins—In Silico studies. Acta Sci. Pol. Technol. Aliment..

[15] He R., Malomo S.A., Alashi A., Girgih A.T., Ju X., Aluko R.E. (2013). Glycinyl-histidinyl-serine (GHS), a novel rapeseed protein-derived peptide has blood pressure-lowering effect in spontaneously hypertensive rats. J. Agric. Food Chem..

[16] Skrzypczak K., Fornal E., Waśko A., Gustaw W. (2019). Effects of probiotic fermentation of selected milk and whey protein preparations on bioactive peptides and technological properties. Ital. J. Food Sci..

[17] Khazaei H., Subedi M., Nickerson M., Martínez-Villaluenga C., Frias J., Vandenberg A. (2019). Seed protein of lentils: Current status, progress, and food applications. Foods.

[18] Hsieh C.-C., Martínez-Villaluenga C., de Lumen B.O., Hernández-Ledesma B. (2018). Updating the research on the chemopreventive and therapeutic role of the peptide lunasin. J. Sci. Food Agric..

[19] Savitha M.N., Siddesha J.M., Suvilesh K.N., Yariswamy M., Vivek H.K., D’Souza C.J., Umashankar M., Vishwanath B.S. (2019). Active-site directed peptide L-Phe-D-His-L-Leu inhibits angiotensin converting enzyme activity and dexamethasone-induced hypertension in rats. Peptides.

[20] Zamyatnin A.A., Borchikov A.S., Vladimirov M.G., Voronina O.L. (2006). The EROP-Moscow oligopeptide database. Nucleic Acids Res..

[21] Heller S.R., McNaught A., Pletnev I., Stein S., Tchekhovskoi D. (2015). InChI, the IUPAC International Chemical Identifier. J. Cheminform..

[22] Kim S., Chen J., Cheng T., Gindulyte A., He J., He S., Li Q., Shoemaker B.A., Thiessen P.A., Yu B. (2019). PubChem 2019 update: Improved access to chemical data. Nucleic Acids Res..

[23] Ashok A., Aparna B.H.S. (2019). Discovery, synthesis, and In vitro evaluation of a novel bioactive peptide for ACE and DPP-IV inhibitory activity. Eur. J. Med. Chem..

[24] Gallego M., Mora L., Toldrá F. (2019). The relevance of dipeptides and tripeptides in the bioactivity and taste of dry-cured ham. Food Prod. Process. Nutr..

[25] Pinciroli M., Aphalo P., Nardo A.E., Añón M.C., Quiroga A.V. (2019). Broken rice as a potential functional ingredient with inhibitory activity of renin and angiotensin-converting enzyme (ACE). Plant Foods Hum. Nutr..

[26] Minkiewicz P., Darewicz M., Iwaniak A., Bucholska J., Starowicz P., Czyrko E. (2016). Internet databases of the properties, enzymatic reactions, and metabolism of small molecules-search options and applications in food science. Int. J. Mol. Sci..

[27] Weininger D. (1988). SMILES, a chemical language and information system. 1. Introduction to methodology and encoding rules. J. Chem. Inf. Comput. Sci..

[28] Peña-Castillo A., Méndez-Lucio O., Owen J.R., Martínez-Mayorga K., Medina-Franco J.L., Engel T., Gasteiger J. (2018). Chemoinformatics in food science. Applied Chemoinformatics: Achievements and Future Opportunities.

[29] Scotti L., Júnior F.J., Ishiki H.M., Ribeiro F.F., Duarte M.C., Santana G.S., Oliveira T.B., Diniz M.D., Quintans-Júnior L.J., Scotti M.T., Grumezescu A.M., Holban A.M. (2018). Computer-aided drug design studies in food chemistry. Natural and Artificial Flavoring Agents and Food Dyes.

[30] Minkiewicz P., Turło M., Iwaniak A., Darewicz M. (2019). Free accessible databases as a source of information about food components and other compounds with anticancer activity–brief review. Molecules.

[31] Iwaniak A., Minkiewicz P., Darewicz M., Hrynkiewicz M. (2016). Food protein-originating peptides as tastants-Physiological, technological, sensory, and bioinformatic approaches. Food Res. Int..

[32] O’Boyle N.M., Banck M., James C.A., Morley C., Vandermeersch T., Hutchison G.R. (2011). Open Babel: An open chemical toolbox. J. Cheminform..

[33] Ortiz-Martinez M., Gonzalez de Mejia E., García-Lara S., Aguilar O., Lopez-Castillo L.M., Otero-Pappatheodorou J.T. (2017). Antiproliferative effect of peptide fractions isolated from a quality protein maize, a white hybrid maize, and their derived peptides on hepatocarcinoma human HepG2 cells. J. Funct. Foods.

[34] Mojica L., Luna-Vital D.A., Gonzalez de Mejia E. (2018). Black bean peptides inhibit glucose uptake in Caco-2 adenocarcinoma cells by blocking the expression and translocation pathway of glucose transporters. Toxicol. Rep..

[35] Yu Z., Fan Y., Zhao W., Ding L., Li J., Liu L. (2018). Novel angiotensin-converting enzyme inhibitory peptides derived from Oncorhynchus mykiss nebulin: Virtual screening and in silico molecular docking study. J. Food Sci..

[36] Bradbury A.F., Smyth D.G. (1991). Peptide amidation. Trends Biochem. Sci..

[37] Lee K.H., Hong S.Y., Oh J.E., Kwon M.Y., Yoon J.H., Lee J.H., Lee B.L., Moon H.M. (1998). Identification and characterization of the antimicrobial peptide corresponding to C-terminal beta-sheet domain of tenecin 1, an antibacterial protein of larvae of *Tenebrio molitor*. Biochem. J..

[38] McDonald A.G., Boyce S., Tipton K.F. (2009). ExplorEnz: The primary source of the IUBMB enzyme list. Nucleic Acids Res..

[39] Jeske L., Placzek S., Schomburg I., Chang A., Schomburg D. (2019). BRENDA in 2019: A European ELIXIR core data resource. Nucleic Acids Res..

[40] Mendez D., Gaulton A., Bento A.P., Chambers J., De Veij M., Félix E., Magariños M.P., Mosquera J.F., Mutowo P., Nowotka M. (2019). ChEMBL: Towards direct deposition of bioassay data. Nucleic Acids Res..

[41] Rawlings N.D., Barrett A.J., Thomas P.D., Huang X., Bateman A., Finn R.D. (2018). The MEROPS database of proteolytic enzymes, their substrates and inhibitors in 2017 and a comparison with peptidases in the PANTHER database. Nucleic Acids Res..

[42] Carvalho-Silva D., Pierleoni A., Pignatelli M., Ong C., Fumis L., Karamanis N., Carmona M., Faulconbridge A., Hercules A., McAuley E. (2019). Open Targets Platform: New developments and updates two years on. Nucleic Acids Res..

[43] Judson R.S., Martin M.T., Egeghy P., Gangwal S., Reif D.M., Kothiya P., Wolf M., Cathey T., Transue T., Smith D. (2012). Aggregating data for computational toxicology applications: The U.S. Environmental Protection Agency (EPA) Aggregated Computational Toxicology Resource (ACToR) system. Int. J. Mol. Sci..

[44] Tomasulo P. (2002). ChemIDplus-super source for chemical and drug information. Med. Ref. Serv. Quart..

[45] Kumar R., Chaudhary K., Sharma M., Nagpal G., Chauhan J.S., Singh S., Gautam A., Raghava G.P. (2015). AHTPDB: A comprehensive platform for analysis and presentation of antihypertensive peptides. Nucleic Acids Res..

[46] Wang G., Li X., Wang Z. (2016). APD3: The antimicrobial peptide database as a tool for research and education. Nucleic Acids Res..

[47] Gilson M.K., Liu T., Baitaluk M., Nicola G., Hwang L., Chong J. (2016). BindingDB in 2015: A public database for medicinal chemistry, computational chemistry and systems pharmacology. Nucleic Acids Res..

[48] Li Q., Zhang C., Chen H., Xue J., Guo X., Liang M., Chen M. (2018). BioPepDB: An integrated data platform for food-derived bioactive peptides. Int. J. Food Sci. Nutr..

[49] Dagan-Wiener A., Di Pizio A., Nissim I., Bahia M.S., Dubovski N., Margulis E., Niv M.Y. (2019). BitterDB: Taste ligands and receptors database in 2019. Nucleic Acids Res..

[50] Van Dorpe S., Bronselaer A., Nielandt J., Stalmans S., Wynendaele E., Audenaert K., Van De Wiele C., Burvenich C., Peremans K., Hsuchou H. (2012). Brainpeps: The blood-brain barrier peptide database. Brain Struct. Funct..

[51] Waghu F.H., Barai R.S., Gurung P., Idicula-Thomas S. (2016). CAMP_R3_: A database on sequences, structures and signatures of antimicrobial peptides. Nucleic Acids Res..

[52] Tyagi A., Tuknait A., Anand P., Gupta S., Sharma M., Mathur D., Joshi A., Singh S., Gautam A., Raghava G.P. (2015). CancerPPD: A database of anticancer peptides and proteins. Nucleic Acids Res..

[53] Hastings J., Owen G., Dekker A., Ennis M., Kale N., Muthukrishnan V., Turner S., Swainston N., Mendes P., Steinbeck C. (2016). ChEBI in 2016: Improved services and an expanding collection of metabolites. Nucleic Acids Res..

[54] Williams A., Tkachenko V. (2014). The Royal Society of Chemistry and the delivery of chemistry data repositories for the community. J. Comput. Aided Mol. Des..

[55] Williams A.J., Grulke C.M., Edwards J., McEachran A.D., Mansouri K., Baker N.C., Patlewicz G., Shah I., Wambaugh J.F., Judson R.S. (2017). The CompTox Chemistry Dashboard: A community data resource for environmental chemistry. J. Cheminform..

[56] Igarashi Y., Eroshkin A., Gramatikova S., Gramatikoff K., Zhang Y., Smith J.W., Osterman A.L., Godzik A. (2007). CutDB: A proteolytic event database. Nucleic Acids Res..

[57] Pirtskhalava M., Gabrielian A., Cruz P., Griggs H.L., Squires R.B., Hurt D.E., Grigolava M., Chubinidze M., Gogoladze G., Vishnepolsky B. (2016). DBAASP v.2: An enhanced database of structure and antimicrobial/cytotoxic activity of natural and synthetic peptides. Nucleic Acids Res..

[58] Wishart D.S., Feunang Y.D., Guo A.C., Lo E.J., Marcu A., Grant J.R., Sajed T., Johnson D., Li C., Sayeeda Z. (2018). DrugBank 5.0: A major update to the DrugBank database for 2018. Nucleic Acids Res..

[59] Gautam A., Chaudhary K., Singh S., Joshi A., Anand P., Tuknait A., Mathur D., Varshney G.C., Raghava G.P. (2014). Hemolytik: A database of experimentally determined hemolytic and non-hemolytic peptides. Nucleic Acids Res..

[60] Wishart D.S., Feunang Y.D., Marcu A., Guo A.C., Liang K., Vázquez-Fresno R., Sajed T., Johnson D., Li C., Karu N. (2018). HMDB 4.0: The human metabolome database for 2018. Nucleic Acids Res..

[61] Kanehisa M., Sato Y., Furumichi M., Morishima K., Tanabe M. (2019). New approach for understanding genome variations in KEGG. Nucleic Acids Res..

[62] Nielsen S.D., Beverly R.L., Qu Y., Dallas D.C. (2017). Milk bioactive peptide database: A comprehensive database of milk protein-derived bioactive peptides and novel visualization. Food Chem..

[63] Haug K., Salek R.M., Conesa P., Hastings J., de Matos P., Rijnbeek M., Mahendraker T., Williams M., Neumann S., Rocca-Serra P. (2013). MetaboLights—An open-access general-purpose repository for metabolomics studies and associated meta-data. Nucleic Acids Res..

[64] Théolier J., Fliss I., Jean J., Hammami R. (2014). MilkAMP: A comprehensive database of antimicrobial peptides of dairy origin. Dairy Sci. Technol..

[65] Ntie-Kang F., Telukunta K.K., Döring K., Simoben C.V., Moumbock A.F.A., Malange Y.I., Njume L.E., Yong J.N., Sippl W., Günther S. (2017). NANPDB: A resource for natural products from northern African sources. J. Nat. Prod..

[66] Wang Y., Wang M., Yin S., Jang R., Wang J., Xue Z., Xu T. (2015). NeuroPep: A comprehensive resource of neuropeptides. Database.

[67] Shtatland T., Guettler D., Kossodo M., Pivovarov M., Weissleder R. (2007). PepBank—A database of peptides based on sequence text mining and public peptide data sources. BMC Bioinform..

[68] Liu F., Baggerman G., Schoofs L., Wets G. (2008). The construction of a bioactive peptide database in *Metazoa*. J. Proteome Res..

[69] Juhász A., Haraszi R., Maulis C. (2015). ProPepper: A curated database for identification and analysis of peptide and immune-responsive epitope composition of cereal grain protein families. Database.

[70] Singh S., Chaudhary K., Dhanda S.K., Bhalla S., Usmani S.S., Gautam A., Tuknait A., Agrawal P., Mathur D., Raghava G.P. (2016). SATPdb: A database of structurally annotated therapeutic peptides. Nucleic Acids Res..

[71] Papadatos G., Davies M., Dedman N., Chambers J., Gaulton A., Siddle J. (2016). SureChEMBL: A large-scale, chemically annotated patent document database. Nucleic Acids Res..

[72] Gfeller D., Michielin O., Zoete V. (2013). SwissSidechain: A molecular and structural database of non-natural sidechains. Nucleic Acids Res..

[73] Sterling T., Irwin J.J. (2015). ZINC 15—ligand discovery for everyone. J. Chem. Inf. Model..

[74] Liu Z.P., Wu L.Y., Wang Y., Zhang X.S., Chen L. (2008). Bridging protein local structures and protein functions. Amino Acids.

[75] Minkiewicz P., Darewicz M., Iwaniak A., Sokołowska J., Starowicz P., Bucholska J., Hrynkiewicz M. (2015). Common amino acid subsequences in a universal proteome-relevance for food science. Int. J. Mol. Sci..

[76] Zamyatnin A.A. (2009). Fragmentomics of natural peptide structures. Biochemistry (Moscow).

[77] Martini S., Conte A., Tagliazucchi D. (2019). Comparative peptidomic profile and bioactivities of cooked beef, pork, chicken and turkey meat after In vitro gastro-intestinal digestion. J. Proteom..

[78] Garcia-Vaquero M., Mora L., Hayes M. (2019). In vitro and in silico approaches to generating and identifying angiotensin-converting enzyme I inhibitory peptides from green macroalga *Ulva lactuca*. Marine Drugs.

[79] Dziuba J., Minkiewicz P., Nałęcz D., Iwaniak A. (1999). Database of biologically active peptide sequences. Nahrung.

[80] Bauchart C., Morzel M., Chambon C., Mirand P.P., Reynès C., Buffière C., Rémond D. (2007). Peptides reproducibly released by in vivo digestion of beef meat and trout flesh in pigs. Br. J. Nutr..

[81] Huang B.-B., Lin H.-C., Chang Y.-W. (2015). Analysis of proteins and potential bioactive peptides from tilapia (*Oreochromis* spp.) processing co-products using proteomic techniques coupled with BIOPEP database. J. Funct. Foods.

[82] Tapal A., Vegarud G.E., Sreedhara A., Tiku P.K. (2019). Nutraceutical protein isolate from pigeon pea (*Cajanus cajan*) milling waste by-product: Functional aspects and digestibility. Food Funct..

[83] Jakubczyk A., Karaś M., Złotek U., Szymanowska U., Baraniak B., Bochnak J. (2019). Peptides obtained from fermented faba bean seeds (*Vicia faba*) as potential inhibitors of an enzyme involved in the pathogenesis of metabolic syndrome. LWT Food Sci. Technol..

[84] Udenigwe C.C., Gong M., Wu S. (2013). In silico analysis of the large and small subunits of cereal RuBisCO as precursors of cryptic bioactive peptides. Process Biochem..

[85] Lin K., Zhang L.W., Han X., Xin L., Meng Z.X., Gong P.M., Cheng D.Y. (2018). Yak milk casein as potential precursor of angiotensin I-converting enzyme inhibitory peptides based on in silico proteolysis. Food Chem..

[86] Dziuba J., Iwaniak A., Minkiewicz P. (2003). Computer-aided characteristics of proteins as potential precursors of bioactive peptides. Polimery.

[87] Minkiewicz P., Dziuba J., Michalska J. (2011). Bovine meat proteins as potential precursors of biologically active peptides—A computational study based on the BIOPEP database. Food Sci. Technol. Int..

[88] Nielsen P.M., Petersen D., Dambmann C. (2001). Improved method for determining food protein degree of hydrolysis. J. Food Sci..

[89] Bastian E.D., Brown R.J. (1996). Plasmin in milk and dairy products: An update. Int. Dairy J..

[90] Huang X.W., Chen L.J., Luo Y.B., Guo H.Y., Ren F.Z. (2011). Purification, characterization, and milk coagulating properties of ginger proteases. J. Dairy Sci..

[91] Yu D., Wang C., Song Y., Zhu J., Zhang X. (2019). Discovery of novel angiotensin-converting enzyme inhibitory peptides from *Todarodes pacificus* and their inhibitory mechanism: In silico and In vitro studies. Int. J. Mol. Sci..

[92] Kandemir-Cavas C., Pérez-Sanchez H., Mert-Ozupek N., Cavas L. (2019). In silico analysis of bioactive peptides in invasive sea grass *Halophila stipulacea*. Cells.

[93] Dziuba J., Niklewicz M., Iwaniak A., Darewicz M., Minkiewicz P. (2005). Structural properties of proteolytic-accessible bioactive fragments of selected animal proteins. Polimery.

[94] Pearson W.R. (2000). Flexible sequence similarity searching with the FASTA3 program package. Methods Mol. Biol..

[95] Nardo A.E., Añón M.C., Parisi G. (2018). Large-scale mapping of bioactive peptides in structural and sequence space. PLoS ONE.

[96] Siani M.A., Weininger D., Blaney J.M. (1994). CHUCKLES: A method for representing and searching peptide and peptoid sequences on both monomer and atomic levels. J. Chem. Inf. Comput. Sci..

[97] Duffy F.J., Verniere M., Devocelle M., Bernard E., Shields D.C., Chubb A.J. (2011). CycloPs: Generating virtual libraries of cyclized and constrained peptides including nonnatural amino acids. J. Chem. Inf. Model..

[98] Minkiewicz P., Iwaniak A., Darewicz M. (2017). Annotation of peptide structures using SMILES and other chemical codes–practical solutions. Molecules.

[99] Hähnke V.D., Kim S., Bolton E.E. (2018). PubChem chemical structure standardization. J. Cheminform..

[100] Brodkorb A., Egger L., Alminger M., Alvito P., Assunção R., Ballance S., Bohn T., Bourlieu-Lacanal C., Boutrou R., Carrière F. (2019). INFOGEST static In vitro simulation of gastrointestinal food digestion. Nat. Protoc..

[101] Minkiewicz P., Dziuba J., Darewicz M., Iwaniak A., Michalska J. (2009). Online programs and databases of peptides and proteolytic enzymes—A brief update for 2007–2008. Food Technol. Biotechnol..

