# Supplementary material for: BIOPEP-UWM Database of Bioactive Peptides: Current Opportunities
Source: Int J Mol Sci. 2019 Nov 27;20(23):5978. doi: 10.3390/ijms20235978 (PMC6928608; doi:10.3390/ijms20235978)
Supplement: Supplementary file 1 [file ijms-20-05978-s001.pdf]

BIOPEP-UWM Database of Bioactive Peptides – Current Opportunities

Piotr Minkiewicz, Anna Iwaniak, Małgorzata Darewicz

Supplement

Homepage

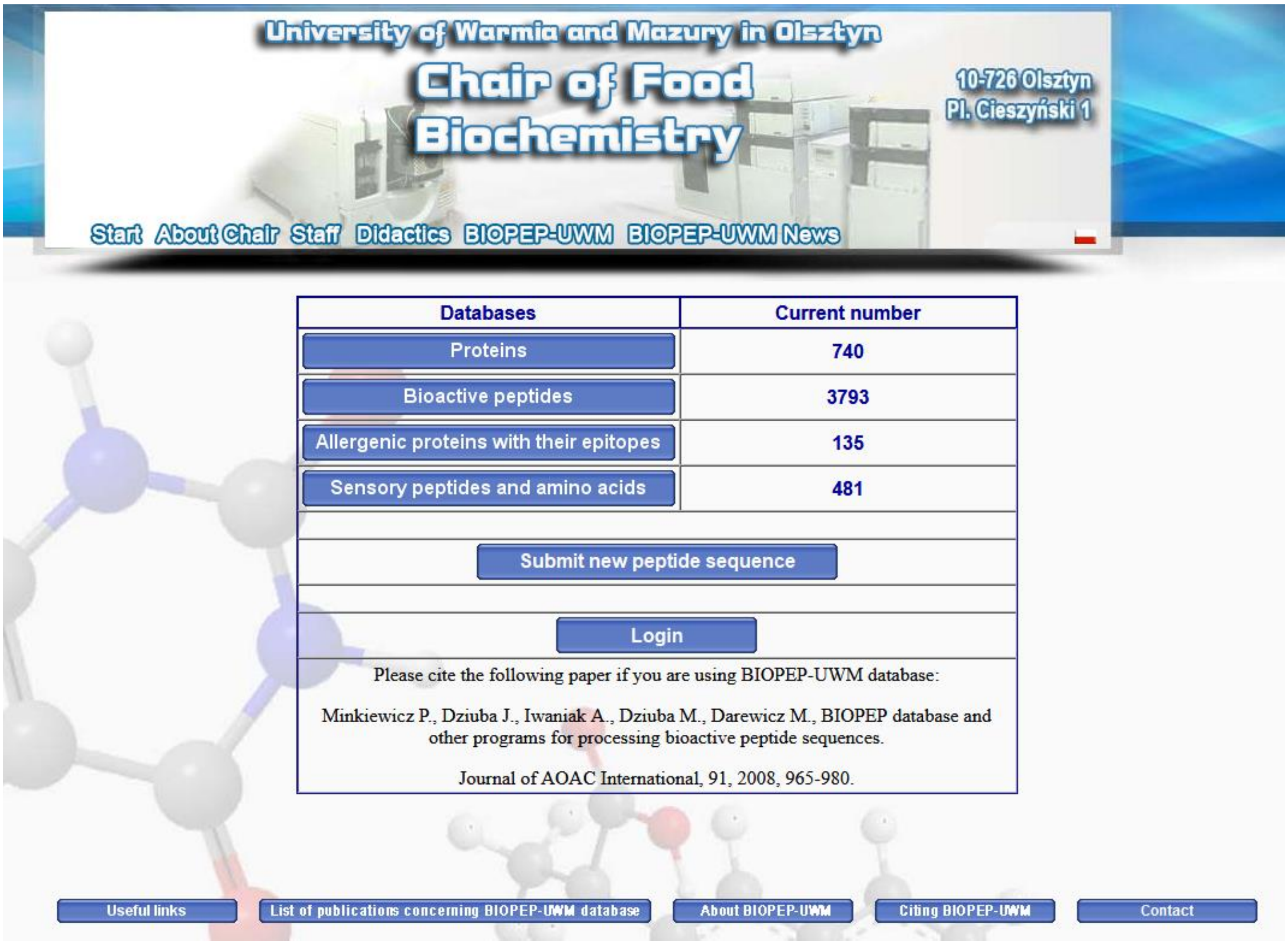

Figure S1. BIOPEP-UWM main page screenshot

“Submit new peptide sequence” tab

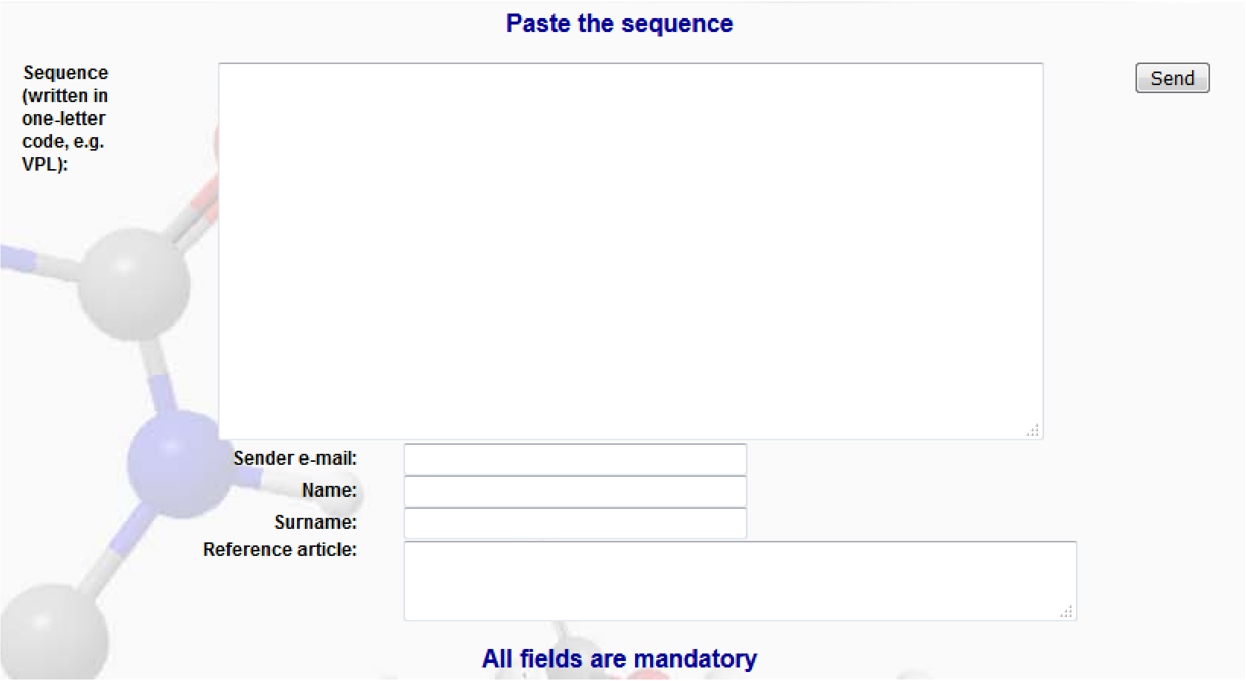

Figure S2. Content of the “Submit new peptide sequence” tab (screenshot)

“Bioactive peptides” tab

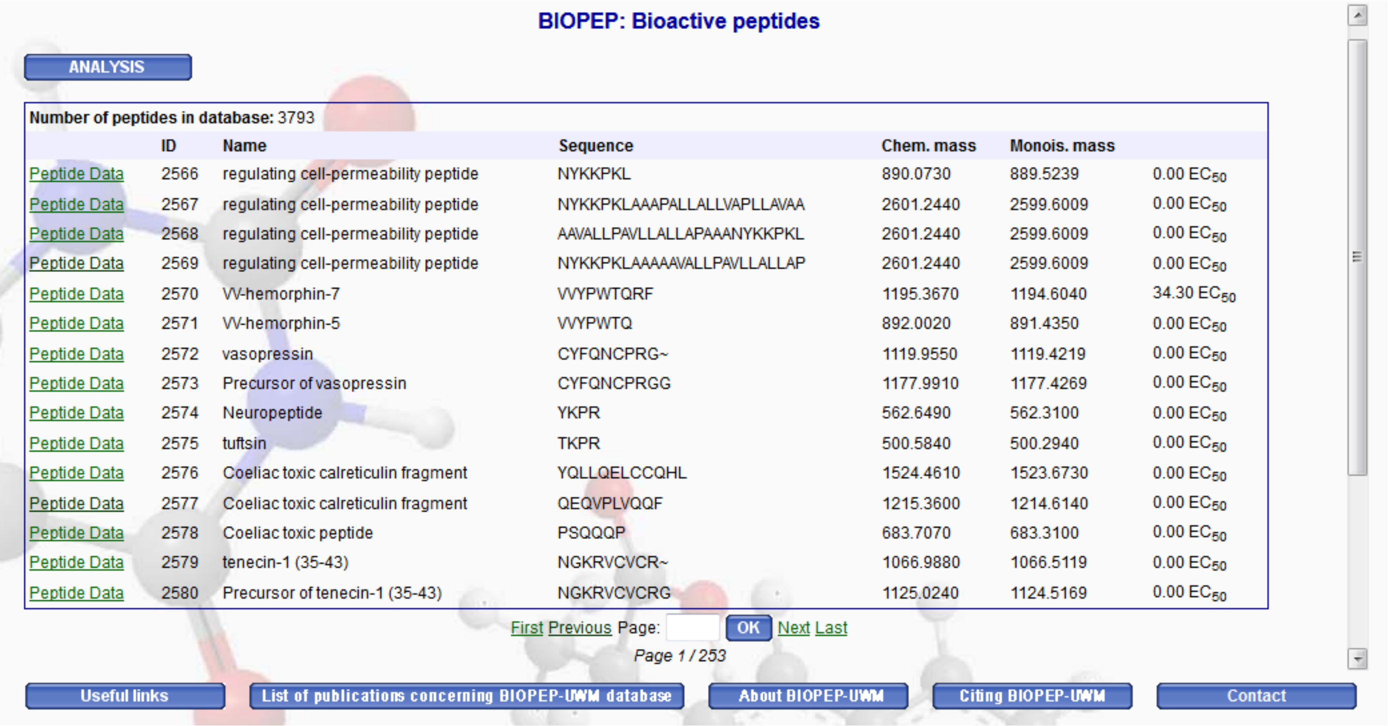

	ID	Name	Sequence	Chem. mass	Monois. mass	
Peptide Data	2566	regulating cell-permeability peptide	NYKKPKL	890.0730	889.5239	0.00 EC_{50}
Peptide Data	2567	regulating cell-permeability peptide	NYKKPKLAAAPALLALLVAPLLAVAA	2601.2440	2599.6009	0.00 EC_{50}
Peptide Data	2568	regulating cell-permeability peptide	AAVALLPAVLLALLAPAAANYKKPKL	2601.2440	2599.6009	0.00 EC_{50}
Peptide Data	2569	regulating cell-permeability peptide	NYKKPKLAAAAAVALLPAVLLALLAP	2601.2440	2599.6009	0.00 EC_{50}
Peptide Data	2570	VV-hemorphin-7	VVYPWTQRF	1195.3670	1194.6040	34.30 EC_{50}
Peptide Data	2571	VV-hemorphin-5	VVYPWTQ	892.0020	891.4350	0.00 EC_{50}
Peptide Data	2572	vasopressin	CYFQNCPRG~	1119.9550	1119.4219	0.00 EC_{50}
Peptide Data	2573	Precursor of vasopressin	CYFQNCPRGG	1177.9910	1177.4269	0.00 EC_{50}
Peptide Data	2574	Neuropeptide	YKPR	562.6490	562.3100	0.00 EC_{50}
Peptide Data	2575	tuftsin	TKPR	500.5840	500.2940	0.00 EC_{50}
Peptide Data	2576	Coeliac toxic calreticulin fragment	YQLLQELCCQHL	1524.4610	1523.6730	0.00 EC_{50}
Peptide Data	2577	Coeliac toxic calreticulin fragment	QEQVPLVQQF	1215.3600	1214.6140	0.00 EC_{50}
Peptide Data	2578	Coeliac toxic peptide	PSQQQP	683.7070	683.3100	0.00 EC_{50}
Peptide Data	2579	tenecin-1 (35-43)	NGKRVCVCR~	1066.9880	1066.5119	0.00 EC_{50}
Peptide Data	2580	Precursor of tenecin-1 (35-43)	NGKRVCVCRG	1125.0240	1124.5169	0.00 EC_{50}

Figure S3. List of bioactive peptides – page 1 screenshot

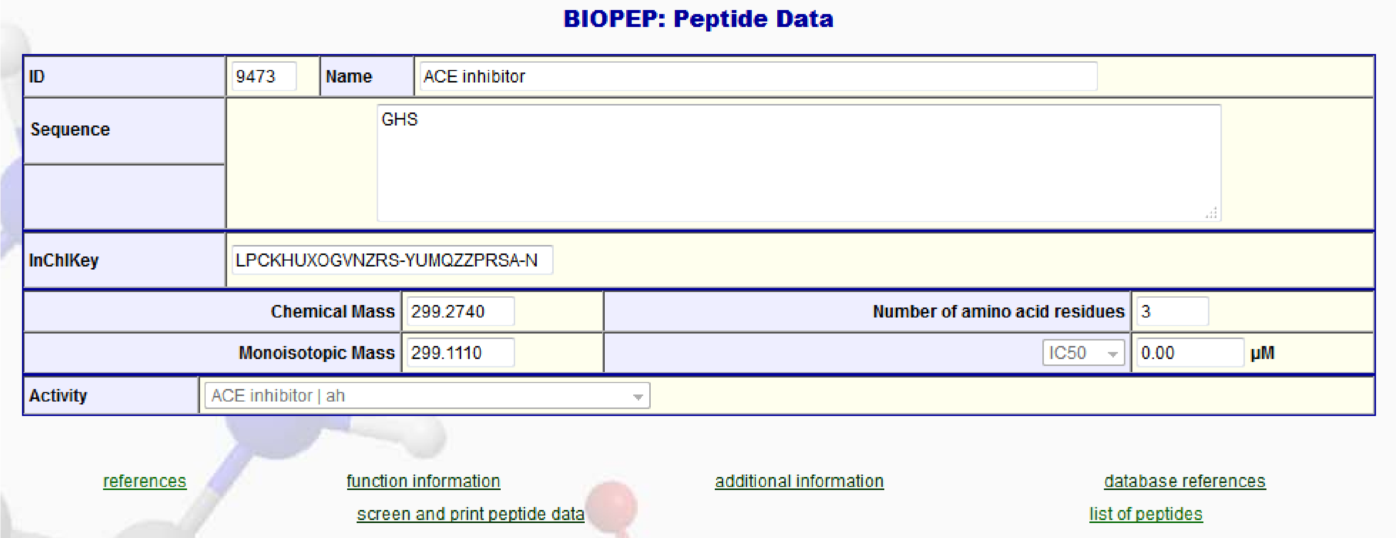

Figure S4. Page of an individual peptide (“Activity” tab) screenshot

Table S1. Content of a page containing representative peptide information (copied from the "Screen and print peptide data" tab)

BIOPEP: Report

ID 9473
Name ACE inhibitor
sequence
GHS

Function:
Inhibitor of Angiotensin-Converting Enzyme (ACE) (EC 3.4.15.1) (MEROPS ID: M02-001)

Number of amino acid residues 3 **Activity code** ah
Activity : ACE inhibitor
Chemical mass 299.2740 **Monoisotopic mass** 299.1110
IC50 : 0.00 µM

Bibliographic data:
Authors
He R., Malomo S. A., Alashi A., Girgih A. T., Ju X., Aluko R. E.
Title
Glycinyl-histidinyl-serine (GHS), a novel rapeseed protein-derived peptide has blood pressure-lowering effect in spontaneously hypertensive rats. J. Agric. Food Chem., 61, 8396-8402, 2013
Year **Editor**
2013 Journal

Additional information:
BIOPEP-UWM database of bioactive peptides

SMILES: NCC(=O)N[C@@H](Cc1c[nH]cn1)C(=O)N[C@@]([H])(CO)C(=O)O

InChI=1S/C11H17N5O5/c12-2-9(18)15-7(1-6-3-13-5-14-6)10(19)16-8(4-17)11(20)21/h3,5,7-8,17H,1-2,4,12H2,(H,13,14)(H,15,18)(H,16,19)(H,20,21)/t7-,8-/m0/s1

InChIKey: LPCKHUXOGVNZRS-YUMQZZPRSA-N

Inhibitor of Renin (EC 3.4.23.15) (MEROPS ID: A01.007) according to the BIOPEP-UWM database of bioactive peptides (ID 9472)

Database reference:
AHTPDB: ID 1053, 2949

BioPepDB: ID biopep00354

BIOPEP-UWM database of bioactive peptides: ID 9472

SATPdb: ID satpdb13065

Search options

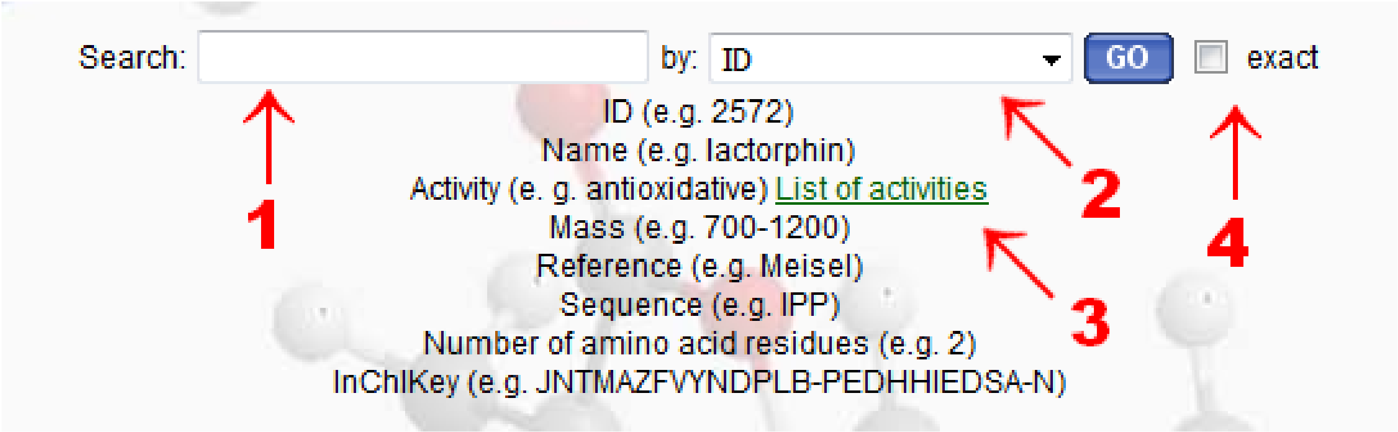

Figure S5. Peptide search options available on the page with a list of peptides (screenshot). 1. Window to paste query, 2. Search option choice menu, 3. Link to the list of activities, 4. Icon of "exact search" option

Analysis

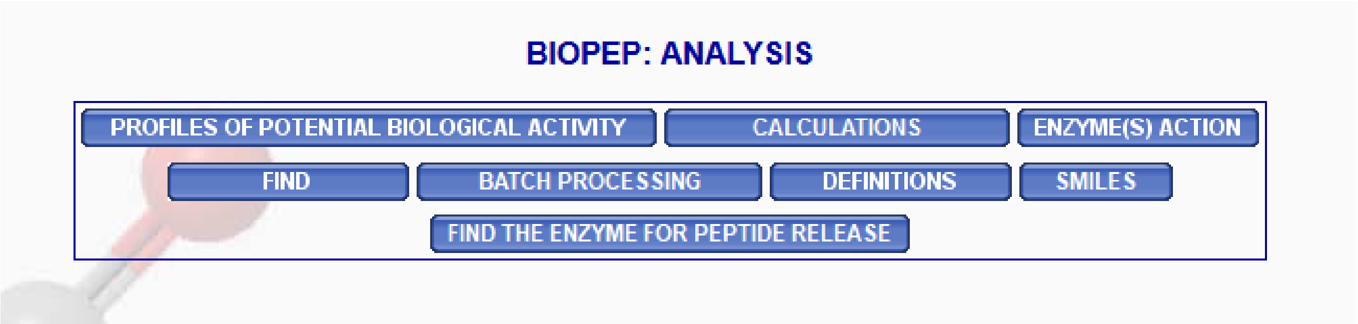

Figure S6. Menu of the "Analysis" tab screenshot

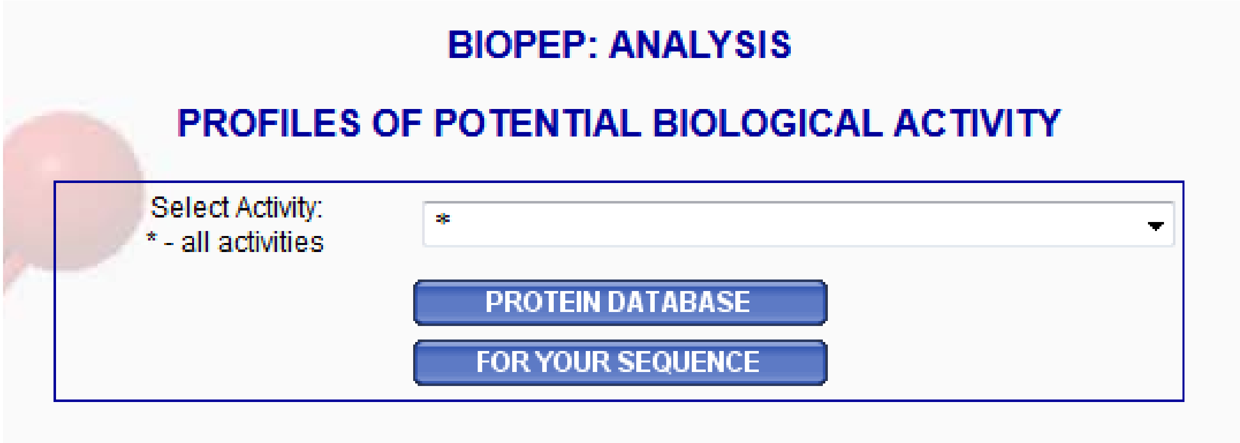

Figure S7. Menu of the "Profiles of potential biological activity" tab

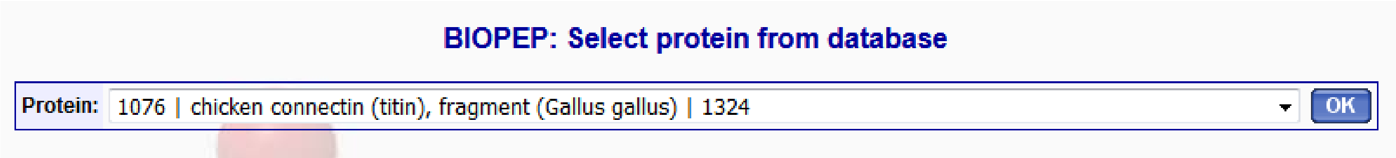

Figure S8. Menu for protein selection from the BIOPEP-UWM database of proteins.

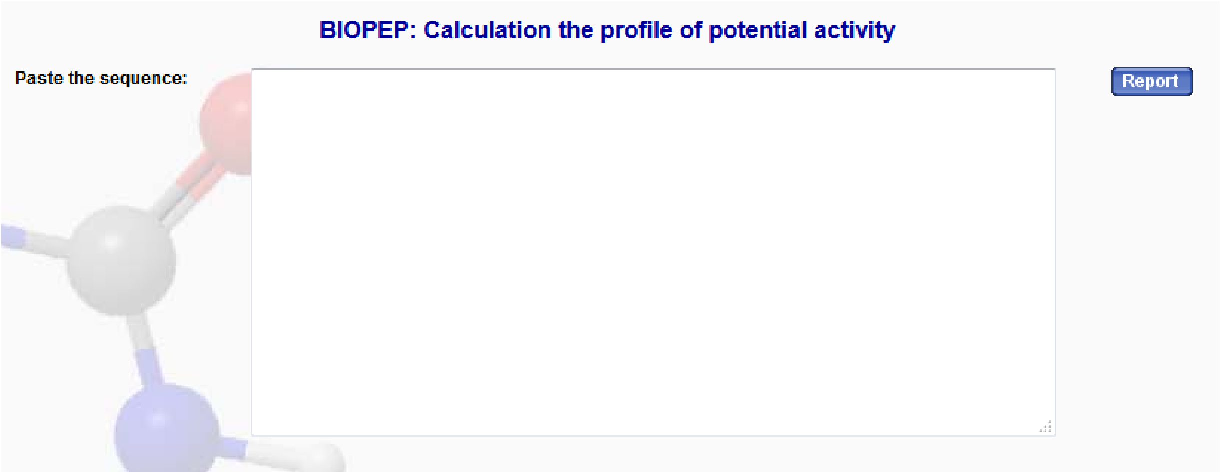

Figure S9. Window for user's sequence input for construction of the profile of potential biological activity of protein or peptide fragments. Analysis can be started by clicking "Report" button.

Table S2. Profile of potential biological activity of an example peptide with the TYHPPPKPPGHP sequence

ID	Name of peptide	Activity	Number	Sequence	Location
3460	Prolyl endopeptidase inhibitor	antiamnestic	1	PG	[9-10]
7601	ACE inhibitor	ACE inhibitor	1	GH	[10-11]
7625	ACE inhibitor	ACE inhibitor	1	PG	[9-10]
7645	ACE inhibitor from porcine myosin (981-983	ACE inhibitor	1	PPK	[5-7]
7810	ACE inhibitor from anchovy and bonito	ACE inhibitor	1	KP	[7-8]
7836	ACE inhibitor	ACE inhibitor	3	PP	[4-5],[5-6],[8-9]
7842	ACE inhibitor	ACE inhibitor	2	HP	[3-4],[11-12]
9087	ACE inhibitor	ACE inhibitor	1	YH	[2-3]
9172	ACE inhibitor	ACE inhibitor	1	PPP	[4-6]
3285	Antithrombotic peptide	antithrombotic	1	PG	[9-10]
8168	peptide derived from κ-casein (109-111)	antithrombotic	1	PPK	[5-7]
2754	peptide regulating the stomach mucosal membrane activity	regulating	1	PG	[9-10]
8218	Antioxidative peptide	antioxidative	1	KP	[7-8]
8219	antioxidative peptide	antioxidative	1	TY	[1-2]
3761	dipeptidyl carboxypeptidase inhibitor	inhibitor	1	PPPK	[4-7]
3170	dipeptidyl peptidase IV inhibitor (DPP IV inhibitor)	dipeptidyl peptidase IV inhibitor	3	PP	[4-5],[5-6],[8-9]
8519	dipeptidyl peptidase IV inhibitor (DPP IV inhibitor)	dipeptidyl peptidase IV inhibitor	1	KP	[7-8]
8520	dipeptidyl peptidase IV inhibitor (DPP IV inhibitor)	dipeptidyl peptidase IV inhibitor	2	HP	[3-4],[11-12]
8653	dipeptidyl peptidase IV inhibitor (DPP IV inhibitor)	dipeptidyl peptidase IV inhibitor	1	PPG	[8-10]
8784	dipeptidyl peptidase IV inhibitor (DPP IV inhibitor)	dipeptidyl peptidase IV inhibitor	1	GH	[10-11]
8855	dipeptidyl peptidase IV inhibitor (DPP IV inhibitor)	dipeptidyl peptidase IV inhibitor	1	PG	[9-10]
8858	dipeptidyl peptidase IV inhibitor (DPP IV inhibitor)	dipeptidyl peptidase IV inhibitor	1	PK	[6-7]
8914	dipeptidyl peptidase IV inhibitor (DPP IV inhibitor)	dipeptidyl peptidase IV inhibitor	1	TY	[1-2]
8937	dipeptidyl peptidase IV inhibitor (DPP IV inhibitor)	dipeptidyl peptidase IV inhibitor	1	YH	[2-3]
9481	DPP-III inhibitor	dipeptidyl peptidase III inhibitor	1	YH	[2-3]
9496	DPP-III inhibitor	dipeptidyl peptidase III inhibitor	2	HP	[3-4],[11-12]

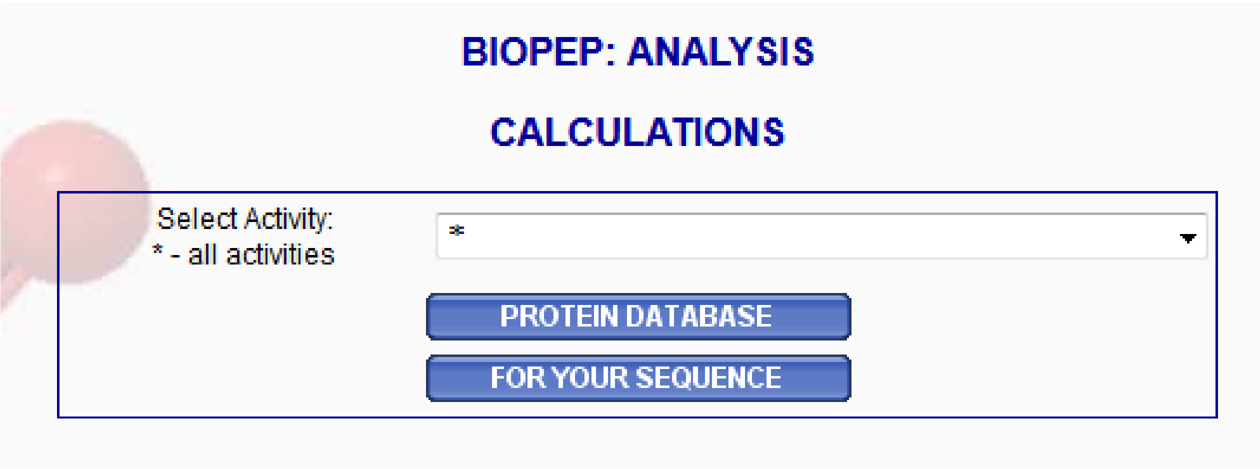

Figure S10. "Calculations" menu screenshot. Menus for "Protein database" and "For your sequence" tabs are the same as presented in Figures S8 and S9, respectively.

Table S3. Results of A calculation of A and B parameters characterizing the quantitative content of bioactive fragments in the TYHPPPKPPGHP sequence. Definitions of A and B parameters are given in Table 5 in the main text.

ID	NAME	Activity	A	B
---	----------------	ACE inhibitor	0.9167	0.021212870691036
---	----------------	antiamnestic	0.0833	
---	----------------	antioxidative	0.1667	
---	----------------	antithrombotic	0.1667	
---	----------------	dipeptidyl peptidase III inhibitor	0.2500	
---	----------------	dipeptidyl peptidase IV inhibitor	1.0000	0.00017156514577392
---	----------------	inhibitor	0.0833	
---	----------------	regulating	0.0833	

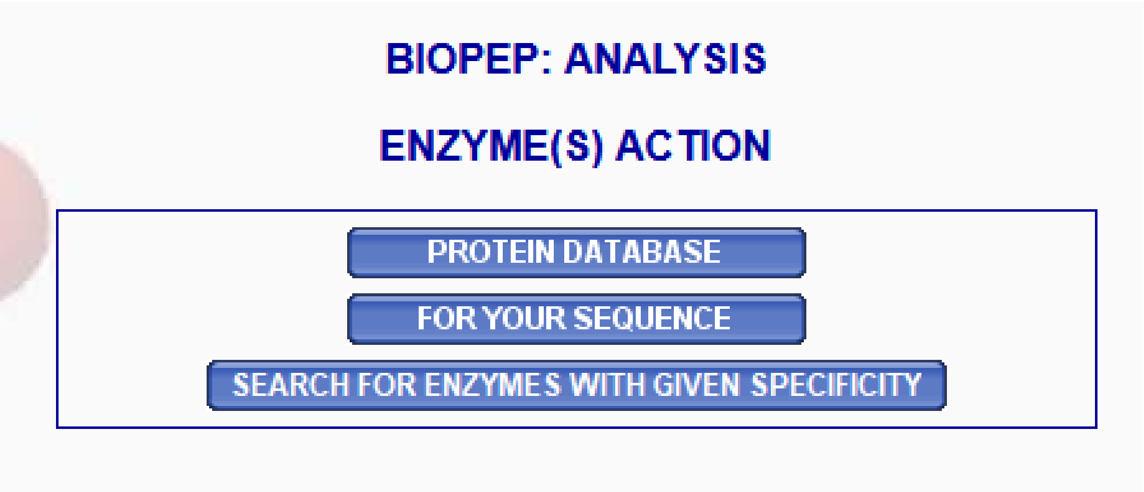

Figure S11. Screenshot of "Enzyme(s) action" menu.

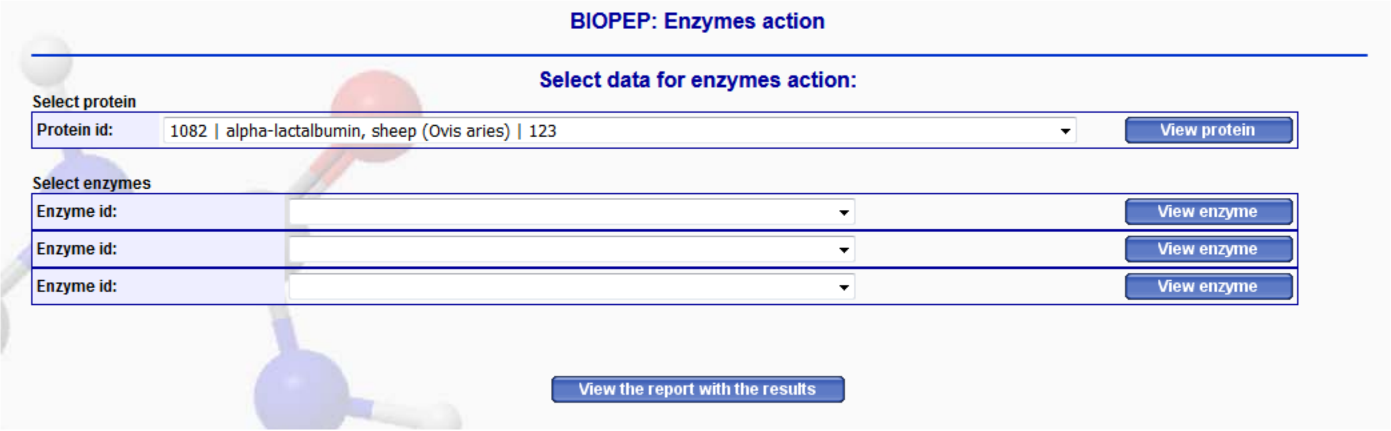

Figure S12. Screenshot of the menu for protein and enzyme selection from the BIOPEP-UWM protein database

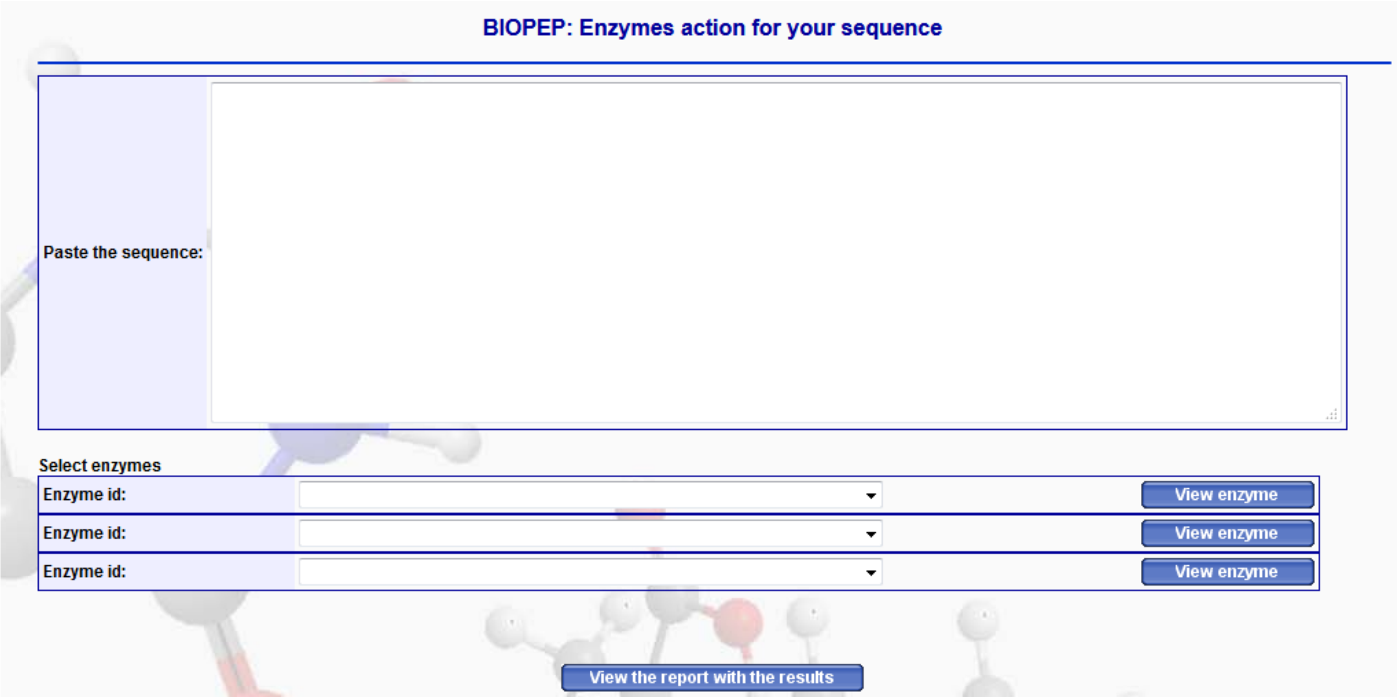

Figure S13. Screenshot of the menu for input of user's protein or peptide and enzyme selection.

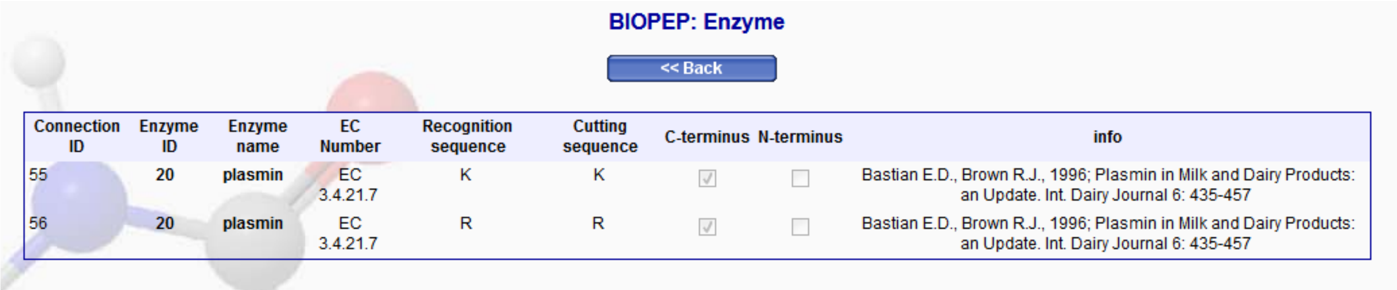

Connection ID	Enzyme ID	Enzyme name	EC Number	Recognition sequence	Cutting sequence	C-terminus	N-terminus	info
55	20	plasmin	EC 3.4.21.7	K	K	☑	☐	Bastian E.D., Brown R.J., 1996; Plasmin in Milk and Dairy Products: an Update. Int. Dairy Journal 6: 435-457
56	20	plasmin	EC 3.4.21.7	R	R	☑	☐	Bastian E.D., Brown R.J., 1996; Plasmin in Milk and Dairy Products: an Update. Int. Dairy Journal 6: 435-457

Figure S14. Screenshot of an example table containing enzyme data (Plasmin; EC 3.4.21.7; MEROPS ID: S01.233).

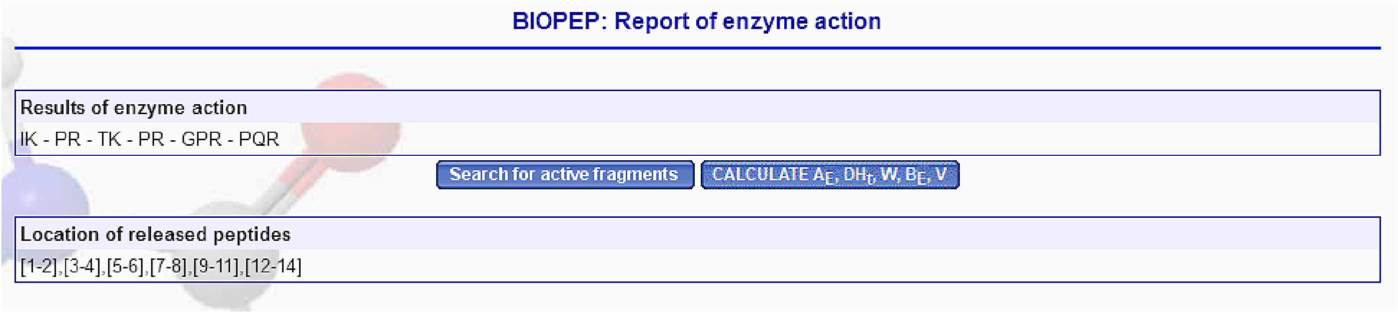

Figure S15. Results of preliminary simulation of hydrolysis of peptide IKPRTKPRGPRPQR using plasmin (see Fig. S14). Buttons: “Search for active fragments” and “Calculate A_E, DH_E, W, B_E, V” serve for further analysis of results.

Table S4. Results of search for active fragments among products of simulated proteolysis of peptide IKPRTKPRGPRPQR using plasmin

No	Peptide ID	Sequence	Location	Name	Function	Activity	Monoisotopic mass	Chemical mass
1	3047	GPR	[9-11]	Antithrombotic peptide	antithrombotic	antithrombotic	328.1740	328.3570
2	3373	PQR	[12-14]	ACE inhibitor from beta-CN	Inhibitor of Angiotensin-Converting Enzyme (ACE) (EC 3.4.15.1) (MEROPS ID: M02-001)	ACE inhibitor	399.2110	399.4360
3	3537	PR	[3-4]	ACE inhibitor	Inhibitor of Angiotensin-Converting Enzyme (ACE) (EC 3.4.15.1) (MEROPS ID: M02-001)	ACE inhibitor	271.1530	271.3050
4	3537	PR	[7-8]	ACE inhibitor	Inhibitor of Angiotensin-Converting Enzyme (ACE) (EC 3.4.15.1) (MEROPS ID: M02-001)	ACE inhibitor	271.1530	271.3050
5	8904	TK	[5-6]	dipeptidyl peptidase IV inhibitor (DPP IV inhibitor)	Inhibitor of Dipeptidyl Peptidase IV (EC 3.4.14.5) (MEROPS ID: S09.003)	dipeptidyl peptidase IV inhibitor	247.1410	247.2790
6	9489	PR	[3-4]	DPP-III inhibitor	Inhibitor of Dipeptidyl peptidase-III (DPP-III) (EC 3.4.14.4) (MEROPS ID: M49.001)	dipeptidyl peptidase III inhibitor	271.1530	271.3050
7	9489	PR	[7-8]	DPP-III inhibitor	Inhibitor of Dipeptidyl peptidase-III (DPP-III) (EC 3.4.14.4) (MEROPS ID: M49.001)	dipeptidyl peptidase III inhibitor	271.1530	271.3050

Table S5. Results of calculation of A_E, DH_E, W, B_E, V parameters characterizing simulated proteolysis of peptide IKPRTKPRGPRPQR using plasmin

DH_t [%]					
38.4615					
No	Activity	A_E	W	B_E	V
1	antithrombotic	0.0714	0.3332	0	
2	ACE inhibitor	0.2143	0.2728	0.035021331688201	0.50010828935411
3	dipeptidyl peptidase IV inhibitor	0.0714	0.1428	0	0
4	dipeptidyl peptidase III inhibitor	0.1429	0.6668	0	0

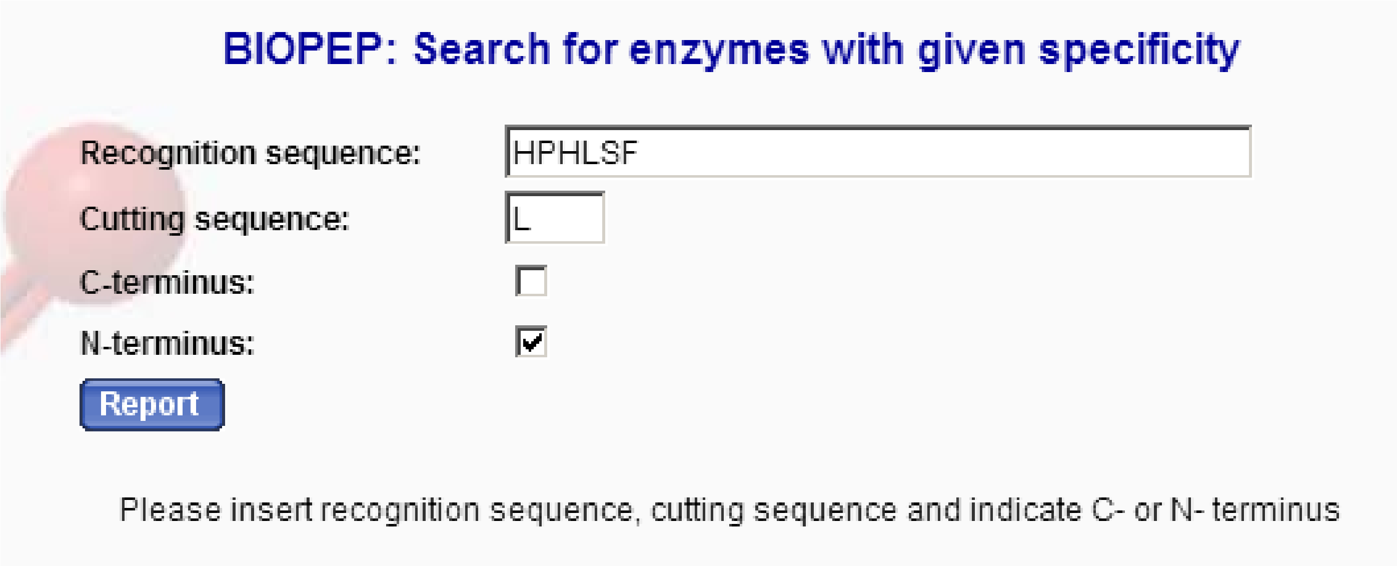

Figure S15. Screenshot of the menu for the "Search for enzymes with given specificity" option

Table S6. Results of search for enzyme with specificity defined as shown in Figure S15.

No	Enzyme name	EC Number	Enzyme ID	Connection ID
1	ginger protease (zingipain)	EC 3.4.22.67	43	140

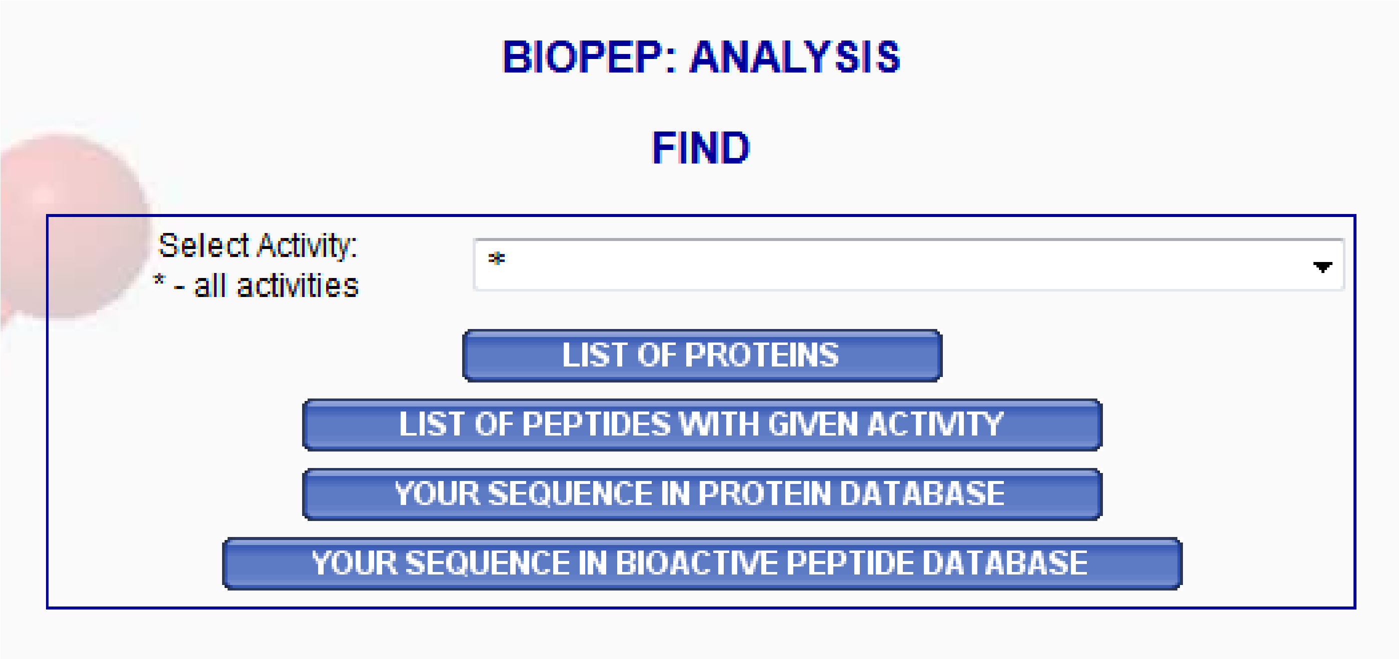

Figure S17. Screenshot of the menu of the "Find" tab.

Table S7. Results from the "Find your sequence in bioactive peptide database" tab. The VPP sequence has been used as a query.

	ID	Name	Sequence
Peptide data	2682	Erythropoietin receptor agonist	DLEGCRLGWVGHCNVWGGDEYTKRTS HSVPPSHKSKLL
Peptide data	3153	Erythropoietin receptor agonist peptide	DVEACGGGWVGHCNYWLRDEYASKPI KQVPPGNHNQPS
Peptide data	3503	ACE inhibitor (bCN fr.80-90)	TPVVVPPFLQP
Peptide data	3524	ACE inhibitor (from bovine beta-CN)	VPP
Peptide data	7664	ACE inhibitor	VVPPA
Peptide data	8308	ACE inhibitor	VVPP
Peptide data	8456	Antioxidant peptide from marine bivalve (Mactra veneriformis)	VAMVPPFET
Peptide data	8661	dipeptidyl peptidase IV inhibitor (DPP IV inhibitor)	VPPFIQPE
Peptide data	9237	ACE inhibitor	LTQTPVVVPPF
Peptide data	9251	ACE inhibitor	VVVPPF
Peptide data	9399	Antioxidative peptide	GQVPP
Peptide data	9409	dipeptidyl peptidase IV inhibitor (DPP IV inhibitor)	VPPFIQPE
Peptide data	9453	Antifungal peptide	FPSHTGMSVPPP

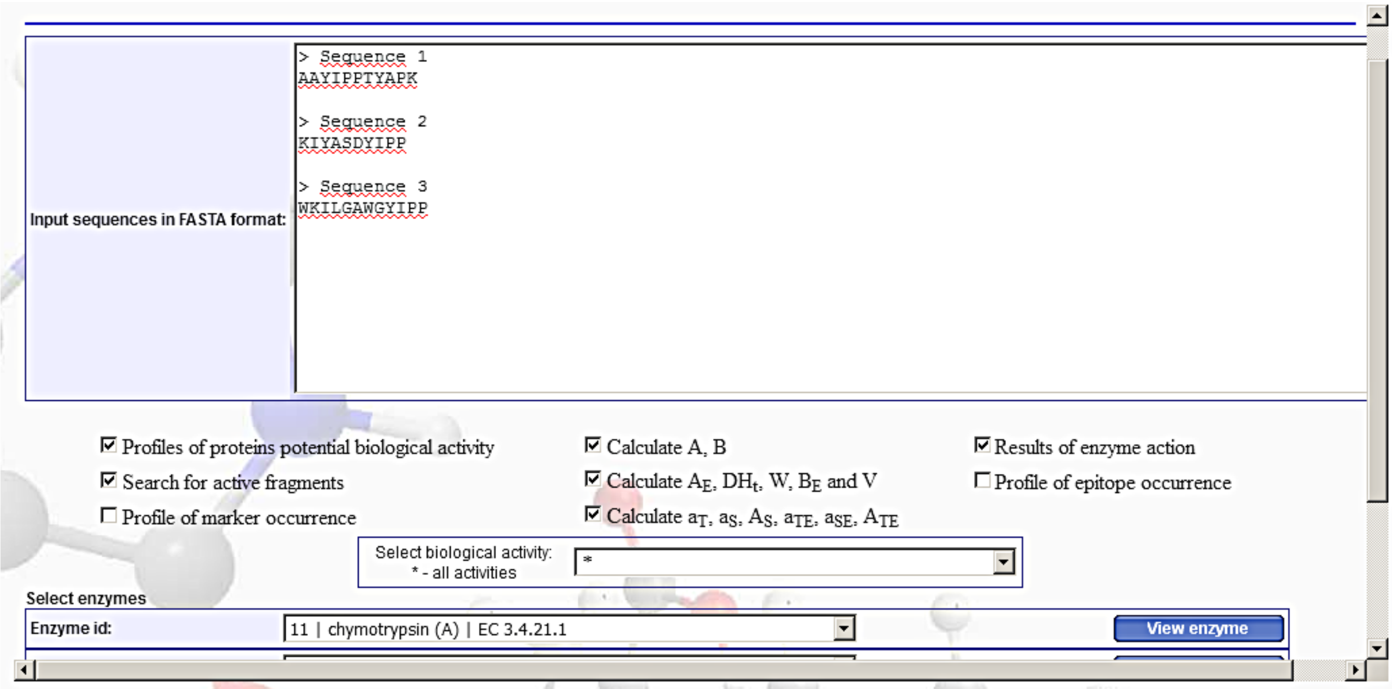

Figure S18. Screenshot of the menu of the "Batch processing" tab. Results for the query sequences displayed in the Figure are presented in Tables S8 and S9.

Table S8. Results of batch processing – step 1. The set of processed sequences was as follows: sequence 1: AAYIPPTYAPK; sequence 2: KIYASDYIPP; sequence 3: WKILGAWGYIPP. The enzyme chymotrypsin A (EC 3.4.21.1; MEROPS ID: S01.001; BIOPEP-UWM ID: 11) was used for proteolysis simulation. Results of calculation of the parameters a_T, a_S, A_S, a_{TE}, a_{SE}, A_{TE} for selected peptides may be displayed by pressing the "View the report with the results" button below the table. Peptides may be selected by clicking icons in the left column in the table showing profile of potential biological activity of fragments or by clicking the "Check all" button to select all peptides.

Protein name: Sequence 1						
Protein sequence: AAYIPPTYAPK						
Profiles of proteins potential biological activity						
As	ID	Name of peptide	Activity	Number	Sequence	Location
☐	3522	ACE inhibitor (from bovine b-CN)	ACE inhibitor	1	IPP	[4-6]
☐	3563	ACE inhibitor	ACE inhibitor	1	AY	[2-3]
☐	7581	ACE inhibitor	ACE inhibitor	1	IP	[4-5]
☐	7584	ACE inhibitor	ACE inhibitor	1	AP	[9-10]
☐	7589	ACE inhibitor	ACE inhibitor	1	YA	[8-9]
☐	7590	ACE inhibitor	ACE inhibitor	1	AA	[1-2]
☐	7833	ACE inhibitor	ACE inhibitor	1	PT	[6-7]
☐	7836	ACE inhibitor	ACE inhibitor	1	PP	[5-6]
☐	7866	peptide from Okara protein	antioxidative	1	AY	[2-3]
☐	8219	antioxidative peptide	antioxidative	1	TY	[7-8]
☐	2886	Opioid peptide	opioid antagonist	1	YIPP	[3-6]
☐	3170	dipeptidyl peptidase IV inhibitor (DPP IV inhibitor)	dipeptidyl peptidase IV inhibitor	1	PP	[5-6]

☐	3177	dipeptidyl peptidase IV inhibitor (DPP IV inhibitor)	dipeptidyl peptidase IV inhibitor	1	AP	[9-10]
☐	8501	Dipeptidyl peptidase IV inhibitor (DPP IV inhibitor)	dipeptidyl peptidase IV inhibitor	1	IP	[4-5]
☐	8637	dipeptidyl peptidase IV inhibitor (DPP IV inhibitor)	dipeptidyl peptidase IV inhibitor	1	AA	[1-2]
☐	8765	dipeptidyl peptidase IV inhibitor (DPP IV inhibitor)	dipeptidyl peptidase IV inhibitor	1	AY	[2-3]
☐	8858	dipeptidyl peptidase IV inhibitor (DPP IV inhibitor)	dipeptidyl peptidase IV inhibitor	1	PK	[10-11]
☐	8863	dipeptidyl peptidase IV inhibitor (DPP IV inhibitor)	dipeptidyl peptidase IV inhibitor	1	PT	[6-7]
☐	8914	dipeptidyl peptidase IV inhibitor (DPP IV inhibitor)	dipeptidyl peptidase IV inhibitor	1	TY	[7-8]
☐	8932	dipeptidyl peptidase IV inhibitor (DPP IV inhibitor)	dipeptidyl peptidase IV inhibitor	1	YA	[8-9]
☐	8938	dipeptidyl peptidase IV inhibitor (DPP IV inhibitor)	dipeptidyl peptidase IV inhibitor	1	YI	[3-4]
☐	9510	DPP-III inhibitor	dipeptidyl peptidase III inhibitor	1	YI	[3-4]
☐	9433	Renin inhibitor	renin inhibitor	1	YA	[8-9]

Calculations

ID	NAME	Activity	A	B
---	----------------	ACE inhibitor	0.7273	0.024405320817811
---	----------------	antioxidative	0.1818	
---	----------------	dipeptidyl peptidase III inhibitor	0.0909	
---	----------------	dipeptidyl peptidase IV inhibitor	0.9091	0.00025834927242301
---	----------------	opioid antagonist	0.0909	
---	----------------	renin inhibitor	0.0909	

Results of enzyme action

AAY - IPPTY - APK

Location of released peptides

[1-3],[4-8],[9-11]

Search for active fragments

No	Peptide ID	Sequence	Location	Name	Function	Activity	Monoisotopic mass	Chemical mass

DH_t [%]

20.0000

CALCULATE A_E, W, B_E and V

No	Activity	A_E	W	B_E	V

Protein name: Sequence 2

Protein sequence: KIYASDYIPP

Profiles of proteins potential biological activity

As	ID	Name of peptide	Activity	Number	Sequence	Location

☐	3383	ACE inhibitor	ACE inhibitor	1	IY	[2-3]
☐	3522	ACE inhibitor (from bovine b-CN)	ACE inhibitor	1	IPP	[8-10]
☐	7581	ACE inhibitor	ACE inhibitor	1	IP	[8-9]
☐	7589	ACE inhibitor	ACE inhibitor	1	YA	[3-4]
☐	7836	ACE inhibitor	ACE inhibitor	1	PP	[9-10]
☐	9072	ACE inhibitor	ACE inhibitor	1	DY	[6-7]
☐	2749	peptide regulating ion flow	regulating	1	DY	[6-7]
☐	7873	peptide from soybean protein isolates: beta-conglycinin and glycinin	antioxidative	1	IY	[2-3]
☐	2886	Opioid peptide	opioid antagonist	1	YIPP	[7-10]
☐	3170	dipeptidyl peptidase IV inhibitor (DPP IV inhibitor)	dipeptidyl peptidase IV inhibitor	1	PP	[9-10]
☐	8501	Dipeptidyl peptidase IV inhibitor (DPP IV inhibitor)	dipeptidyl peptidase IV inhibitor	1	IP	[8-9]
☐	8762	dipeptidyl peptidase IV inhibitor (DPP IV inhibitor)	dipeptidyl peptidase IV inhibitor	1	AS	[4-5]
☐	8812	dipeptidyl peptidase IV inhibitor (DPP IV inhibitor)	dipeptidyl peptidase IV inhibitor	1	KI	[1-2]
☐	8932	dipeptidyl peptidase IV inhibitor (DPP IV inhibitor)	dipeptidyl peptidase IV inhibitor	1	YA	[3-4]
☐	8938	dipeptidyl peptidase IV inhibitor (DPP IV inhibitor)	dipeptidyl peptidase IV inhibitor	1	YI	[7-8]
☐	9510	DPP-III inhibitor	dipeptidyl peptidase III inhibitor	1	YI	[7-8]
☐	9433	Renin inhibitor	renin inhibitor	1	YA	[3-4]

Calculations

ID	NAME	Activity	A	B
---	----------------	ACE inhibitor	0.6000	0.069605669692626
---	----------------	antioxidative	0.1000	
---	----------------	dipeptidyl peptidase III inhibitor	0.1000	
---	----------------	dipeptidyl peptidase IV inhibitor	0.6000	0.00026096728544077
---	----------------	opioid antagonist	0.1000	
---	----------------	regulating	0.1000	
---	----------------	renin inhibitor	0.1000	

Results of enzyme action

KIY - ASDY - IPP

Location of released peptides

[1-3],[4-7],[8-10]

Search for active fragments

No	Peptide ID	Sequence	Location	Name	Function	Activity	Monoisotopic mass	Chemical mass

1	3522	IPP	[8-10]	ACE inhibitor (from bovine b-CN)		ACE inhibitor	325.1880	325.3940

DH_t [%]
22.2222
CALCULATE A_E, W, B_E and V

No	Activity	A_E	W	B_E	V
1	ACE inhibitor	0.1000	0.1667	0.02	0.28733291538345

Protein name: Sequence 3
Protein sequence: WKILGAWGYIPP
Profiles of proteins potential biological activity

As	ID	Name of peptide	Activity	Number	Sequence	Location
☐	3522	ACE inhibitor (from bovine b-CN)	ACE inhibitor	1	IPP	[10-12]
☐	3532	ACE inhibitor	ACE inhibitor	1	GY	[8-9]
☐	7543	ACE inhibitor	ACE inhibitor	1	AW	[6-7]
☐	7581	ACE inhibitor	ACE inhibitor	1	IP	[10-11]
☐	7598	ACE inhibitor	ACE inhibitor	1	GA	[5-6]
☐	7613	ACE inhibitor	ACE inhibitor	1	WG	[7-8]
☐	7619	ACE inhibitor	ACE inhibitor	1	LG	[4-5]
☐	7836	ACE inhibitor	ACE inhibitor	1	PP	[11-12]
☐	9079	ACE inhibitor	ACE inhibitor	1	IL	[3-4]
☐	8323	Glucose uptake stimulating peptide	stimulating	1	IL	[3-4]
☐	8460	Antioxidant peptide from marine bivalve (Mactra veneriformis)	antioxidative	1	AW	[6-7]
☐	9082	Antioxidative peptide	antioxidative	1	WG	[7-8]
☐	3201	chymotrypsin inhibitor	inhibitor	1	GAW	[5-7]
☐	2886	Opioid peptide	opioid antagonist	1	YIPP	[9-12]
☐	3170	dipeptidyl peptidase IV inhibitor (DPP IV inhibitor)	dipeptidyl peptidase IV inhibitor	1	PP	[11-12]
☐	8501	Dipeptidyl peptidase IV inhibitor (DPP IV inhibitor)	dipeptidyl peptidase IV inhibitor	1	IP	[10-11]
☐	8524	dipeptidyl peptidase IV inhibitor (DPP IV inhibitor)	dipeptidyl peptidase IV inhibitor	1	GA	[5-6]
☐	8676	dipeptidyl peptidase IV inhibitor (DPP IV inhibitor)	dipeptidyl peptidase IV inhibitor	1	WK	[1-2]
☐	8695	dipeptidyl peptidase IV inhibitor (DPP IV inhibitor)	dipeptidyl peptidase IV inhibitor	1	AW	[6-7]
☐	8697	dipeptidyl peptidase IV inhibitor (DPP IV inhibitor)	dipeptidyl peptidase IV inhibitor	1	WG	[7-8]
☐	8788	dipeptidyl peptidase IV inhibitor (DPP IV inhibitor)	dipeptidyl peptidase IV inhibitor	1	GY	[8-9]
☐	8802	dipeptidyl peptidase IV inhibitor	dipeptidyl peptidase IV	1	IL	[3-4]

		(DPP IV inhibitor)	inhibitor			
☐	8812	dipeptidyl peptidase IV inhibitor (DPP IV inhibitor)	dipeptidyl peptidase IV inhibitor	1	KI	[2-3]
☐	8938	dipeptidyl peptidase IV inhibitor (DPP IV inhibitor)	dipeptidyl peptidase IV inhibitor	1	YI	[9-10]
☐	9510	DPP-III inhibitor	dipeptidyl peptidase III inhibitor	1	YI	[9-10]

Calculations

ID	NAME	Activity	A	B
---	----------------	ACE inhibitor	0.7500	0.027619641874223
---	----------------	antioxidative	0.1667	
---	----------------	dipeptidyl peptidase III inhibitor	0.0833	
---	----------------	dipeptidyl peptidase IV inhibitor	0.8333	0.0022700178938607
---	----------------	inhibitor	0.0833	
---	----------------	opioid antagonist	0.0833	
---	----------------	stimulating	0.0833	

Results of enzyme action

W - KIL - GAW - GY - IPP

Location of released peptides

[1-1],[2-4],[5-7],[8-9],[10-12]

Search for active fragments

No	Peptide ID	Sequence	Location	Name	Function	Activity	Monoisotopic mass	Chemical mass
1	3201	GAW	[5-7]	chymotrypsin inhibitor		inhibitor	332.1370	332.3440
2	3522	IPP	[10-12]	ACE inhibitor (from bovine b-CN)		ACE inhibitor	325.1880	325.3940
3	3532	GY	[8-9]	ACE inhibitor	Inhibitor of Angiotensin-Converting Enzyme (ACE) (EC 3.4.15.1) (MEROPS ID: XM02-001)	ACE inhibitor	238.0840	238.2220
4	8788	GY	[8-9]	dipeptidyl peptidase IV inhibitor (DPP IV inhibitor)	Inhibitor of Dipeptidyl Peptidase IV (EC 3.4.14.5) (MEROPS ID: S09.003)	dipeptidyl peptidase IV inhibitor	238.0840	238.2220

DH_t [%]

36.3636

CALCULATE A_E, W, B_E and V

No	Activity	A_E	W	B_E	V
1	inhibitor	0.0833	1.0000	0	
2	ACE inhibitor	0.1667	0.2223	0.033730158730159	1.2212380914916
3	dipeptidyl peptidase IV inhibitor	0.0833	0.1000	0	0

View the report with the results

Table S9. Final results of batch processing. The set of processed sequences was as follows: sequence 1: AAYIPPTYAPK; sequence 2: KIYASDYIPP; sequence 3: WKILGAWGYIPP. The enzyme chymotrypsin A (EC 3.4.21.1; MEROPS ID: S01.001; BIOPEP-UWM ID: 11) was used for proteolysis simulation. Results of calculation of the parameters a_T, a_S, A_S, a_{TE}, a_{SE}, A_{TE} for selected peptides may be displayed by pressing the “View the report with the results” button below the table with results of step 1 (Table S8).

Protein name: Sequence 1					
Protein sequence: AAYIPPTYAPK					
Profiles of proteins potential biological activity					
ID	Name of peptide	Activity	Number	Sequence	Location
3522	ACE inhibitor (from bovine b-CN)	ACE inhibitor	1	IPP	[4-6]
3563	ACE inhibitor	ACE inhibitor	1	AY	[2-3]
7581	ACE inhibitor	ACE inhibitor	1	IP	[4-5]
7584	ACE inhibitor	ACE inhibitor	1	AP	[9-10]
7589	ACE inhibitor	ACE inhibitor	1	YA	[8-9]
7590	ACE inhibitor	ACE inhibitor	1	AA	[1-2]
7833	ACE inhibitor	ACE inhibitor	1	PT	[6-7]
7836	ACE inhibitor	ACE inhibitor	1	PP	[5-6]
7866	peptide from Okara protein	antioxidative	1	AY	[2-3]
8219	antioxidative peptide	antioxidative	1	TY	[7-8]
2886	Opioid peptide	opioid antagonist	1	YIPP	[3-6]
3170	dipeptidyl peptidase IV inhibitor (DPP IV inhibitor)	dipeptidyl peptidase IV inhibitor	1	PP	[5-6]
3177	dipeptidyl peptidase IV inhibitor (DPP IV inhibitor)	dipeptidyl peptidase IV inhibitor	1	AP	[9-10]
8501	Dipeptidyl peptidase IV inhibitor (DPP IV inhibitor)	dipeptidyl peptidase IV inhibitor	1	IP	[4-5]
8637	dipeptidyl peptidase IV inhibitor (DPP IV inhibitor)	dipeptidyl peptidase IV inhibitor	1	AA	[1-2]
8765	dipeptidyl peptidase IV inhibitor (DPP IV inhibitor)	dipeptidyl peptidase IV inhibitor	1	AY	[2-3]
8858	dipeptidyl peptidase IV inhibitor (DPP IV inhibitor)	dipeptidyl peptidase IV inhibitor	1	PK	[10-11]
8863	dipeptidyl peptidase IV inhibitor (DPP IV inhibitor)	dipeptidyl peptidase IV inhibitor	1	PT	[6-7]
8914	dipeptidyl peptidase IV inhibitor (DPP IV inhibitor)	dipeptidyl peptidase IV inhibitor	1	TY	[7-8]
8932	dipeptidyl peptidase IV inhibitor (DPP IV inhibitor)	dipeptidyl peptidase IV inhibitor	1	YA	[8-9]
8938	dipeptidyl peptidase IV inhibitor (DPP IV inhibitor)	dipeptidyl peptidase IV inhibitor	1	YI	[3-4]
9510	DPP-III inhibitor	dipeptidyl peptidase III inhibitor	1	YI	[3-4]
9433	Renin inhibitor	renin inhibitor	1	YA	[8-9]
Calculations					

ID	NAME	Activity	A	B
---	----------------	ACE inhibitor	0.7273	0.024405320817811
---	----------------	antioxidative	0.1818	
---	----------------	dipeptidyl peptidase III inhibitor	0.0909	
---	----------------	dipeptidyl peptidase IV inhibitor	0.9091	0.00025834927242301
---	----------------	opioid antagonist	0.0909	
---	----------------	renin inhibitor	0.0909	

Results of enzyme action
AAY - IPPTY - APK
Location of released peptides
[1-3],[4-8],[9-11]
Search for active fragments

No	Peptide ID	Sequence	Location	Name	Function	Activity	Monoisotopic mass	Chemical mass

DH_t [%]
20.0000
CALCULATE A_E, W, B_E and V

No	Activity	A_E	W	B_E	V

Protein name: Sequence 2
Protein sequence: KIYASDYIPP
Profiles of proteins potential biological activity

ID	Name of peptide	Activity	Number	Sequence	Location
3383	ACE inhibitor	ACE inhibitor	1	IY	[2-3]
3522	ACE inhibitor (from bovine b-CN)	ACE inhibitor	1	IPP	[8-10]
7581	ACE inhibitor	ACE inhibitor	1	IP	[8-9]
7589	ACE inhibitor	ACE inhibitor	1	YA	[3-4]
7836	ACE inhibitor	ACE inhibitor	1	PP	[9-10]
9072	ACE inhibitor	ACE inhibitor	1	DY	[6-7]
2749	peptide regulating ion flow	regulating	1	DY	[6-7]
7873	peptide from soybean protein isolates: beta-conglycinin and glycinin	antioxidative	1	IY	[2-3]
2886	Opioid peptide	opioid antagonist	1	YIPP	[7-10]
3170	dipeptidyl peptidase IV inhibitor (DPP IV inhibitor)	dipeptidyl peptidase IV inhibitor	1	PP	[9-10]
8501	Dipeptidyl peptidase IV inhibitor (DPP IV inhibitor)	dipeptidyl peptidase IV inhibitor	1	IP	[8-9]
8762	dipeptidyl peptidase IV inhibitor (DPP IV inhibitor)	dipeptidyl peptidase IV inhibitor	1	AS	[4-5]
8812	dipeptidyl peptidase IV inhibitor (DPP IV inhibitor)	dipeptidyl peptidase IV inhibitor	1	KI	[1-2]
8932	dipeptidyl peptidase IV inhibitor	dipeptidyl peptidase IV	1	YA	[3-4]

	(DPP IV inhibitor)	inhibitor			
8938	dipeptidyl peptidase IV inhibitor (DPP IV inhibitor)	dipeptidyl peptidase IV inhibitor	1	YI	[7-8]
9510	DPP-III inhibitor	dipeptidyl peptidase III inhibitor	1	YI	[7-8]
9433	Renin inhibitor	renin inhibitor	1	YA	[3-4]

Calculations

ID	NAME	Activity	A	B
---	----------------	ACE inhibitor	0.6000	0.069605669692626
---	----------------	antioxidative	0.1000	
---	----------------	dipeptidyl peptidase III inhibitor	0.1000	
---	----------------	dipeptidyl peptidase IV inhibitor	0.6000	0.00026096728544077
---	----------------	opioid antagonist	0.1000	
---	----------------	regulating	0.1000	
---	----------------	renin inhibitor	0.1000	

Results of enzyme action

KIY - ASDY - IPP

Location of released peptides

[1-3],[4-7],[8-10]

Search for active fragments

No	Peptide ID	Sequence	Location	Name	Function	Activity	Monoisotopic mass	Chemical mass
1	3522	IPP	[8-10]	ACE inhibitor (from bovine b-CN)		ACE inhibitor	325.1880	325.3940

DH_t [%]

22.2222

CALCULATE A_E, W, B_E and V

No	Activity	A_E	W	B_E	V
1	ACE inhibitor	0.1000	0.1667	0.02	0.28733291538345

Protein name: Sequence 3

Protein sequence: WKILGAWGYIPP

Profiles of proteins potential biological activity

ID	Name of peptide	Activity	Number	Sequence	Location
3522	ACE inhibitor (from bovine b-CN)	ACE inhibitor	1	IPP	[10-12]
3532	ACE inhibitor	ACE inhibitor	1	GY	[8-9]
7543	ACE inhibitor	ACE inhibitor	1	AW	[6-7]
7581	ACE inhibitor	ACE inhibitor	1	IP	[10-11]
7598	ACE inhibitor	ACE inhibitor	1	GA	[5-6]

7613	ACE inhibitor	ACE inhibitor	1	WG	[7-8]
7619	ACE inhibitor	ACE inhibitor	1	LG	[4-5]
7836	ACE inhibitor	ACE inhibitor	1	PP	[11-12]
9079	ACE inhibitor	ACE inhibitor	1	IL	[3-4]
8323	Glucose uptake stimulating peptide	stimulating	1	IL	[3-4]
8460	Antioxidant peptide from marine bivalve (Mactra veneriformis)	antioxidative	1	AW	[6-7]
9082	Antioxidative peptide	antioxidative	1	WG	[7-8]
3201	chymotrypsin inhibitor	inhibitor	1	GAW	[5-7]
2886	Opioid peptide	opioid antagonist	1	YIPP	[9-12]
3170	dipeptidyl peptidase IV inhibitor (DPP IV inhibitor)	dipeptidyl peptidase IV inhibitor	1	PP	[11-12]
8501	Dipeptidyl peptidase IV inhibitor (DPP IV inhibitor)	dipeptidyl peptidase IV inhibitor	1	IP	[10-11]
8524	dipeptidyl peptidase IV inhibitor (DPP IV inhibitor)	dipeptidyl peptidase IV inhibitor	1	GA	[5-6]
8676	dipeptidyl peptidase IV inhibitor (DPP IV inhibitor)	dipeptidyl peptidase IV inhibitor	1	WK	[1-2]
8695	dipeptidyl peptidase IV inhibitor (DPP IV inhibitor)	dipeptidyl peptidase IV inhibitor	1	AW	[6-7]
8697	dipeptidyl peptidase IV inhibitor (DPP IV inhibitor)	dipeptidyl peptidase IV inhibitor	1	WG	[7-8]
8788	dipeptidyl peptidase IV inhibitor (DPP IV inhibitor)	dipeptidyl peptidase IV inhibitor	1	GY	[8-9]
8802	dipeptidyl peptidase IV inhibitor (DPP IV inhibitor)	dipeptidyl peptidase IV inhibitor	1	IL	[3-4]
8812	dipeptidyl peptidase IV inhibitor (DPP IV inhibitor)	dipeptidyl peptidase IV inhibitor	1	KI	[2-3]
8938	dipeptidyl peptidase IV inhibitor (DPP IV inhibitor)	dipeptidyl peptidase IV inhibitor	1	YI	[9-10]
9510	DPP-III inhibitor	dipeptidyl peptidase III inhibitor	1	YI	[9-10]

Calculations

ID	NAME	Activity	A	B
---	----------------	ACE inhibitor	0.7500	0.027619641874223
---	----------------	antioxidative	0.1667	
---	----------------	dipeptidyl peptidase III inhibitor	0.0833	
---	----------------	dipeptidyl peptidase IV inhibitor	0.8333	0.0022700178938607
---	----------------	inhibitor	0.0833	
---	----------------	opioid antagonist	0.0833	
---	----------------	stimulating	0.0833	

Results of enzyme action

W - KIL - GAW - GY - IPP

Location of released peptides

[1-1],[2-4],[5-7],[8-9],[10-12]

Search for active fragments

No	Peptide ID	Sequence	Location	Name	Function	Activity	Monoisotopic mass	Chemical mass
1	3201	GAW	[5-7]	chymotrypsin inhibitor		inhibitor	332.1370	332.3440
2	3522	IPP	[10-12]	ACE inhibitor (from bovine b-CN)		ACE inhibitor	325.1880	325.3940
3	3532	GY	[8-9]	ACE inhibitor	Inhibitor of Angiotensin-Converting Enzyme (ACE) (EC 3.4.15.1) (MEROPS ID: XM02-001)	ACE inhibitor	238.0840	238.2220
4	8788	GY	[8-9]	dipeptidyl peptidase IV inhibitor (DPP IV inhibitor)	Inhibitor of Dipeptidyl Peptidase IV (EC 3.4.14.5) (MEROPS ID: S09.003)	dipeptidyl peptidase IV inhibitor	238.0840	238.2220

DH_t [%]
36.3636

CALCULATE A_E, W, B_E and V

No	Activity	A_E	W	B_E	V
1	inhibitor	0.0833	1.0000	0	
2	ACE inhibitor	0.1667	0.2223	0.033730158730159	1.2212380914916
3	dipeptidyl peptidase IV inhibitor	0.0833	0.1000	0	0

CALCULATE a_T, a_S, A_S, a_{TE}, a_{SE}, A_{TE}

ID	Sequence	Activity	a_T	a_S	A_S	a_{TE}	a_{SE}	A_{TE}
3522	IPP	ACE inhibitor	3	1.000	0.091	6	2.000	0.182
3563	AY	ACE inhibitor	1	0.333	0.091	0	0.000	0.000
7581	IP	ACE inhibitor	3	1.000	0.091	0	0.000	0.000
7584	AP	ACE inhibitor	1	0.333	0.091	0	0.000	0.000
7589	YA	ACE inhibitor	2	0.667	0.095	0	0.000	0.000
7590	AA	ACE inhibitor	1	0.333	0.091	0	0.000	0.000
7833	PT	ACE inhibitor	1	0.333	0.091	0	0.000	0.000
7836	PP	ACE inhibitor	3	1.000	0.091	0	0.000	0.000
7866	AY	antioxidative	1	0.333	0.091	0	0.000	0.000
8219	TY	antioxidative	1	0.333	0.091	0	0.000	0.000
2886	YIPP	opioid antagonist	3	1.000	0.091	0	0.000	0.000
3170	PP	dipeptidyl peptidase IV inhibitor	3	1.000	0.091	0	0.000	0.000
3177	AP	dipeptidyl peptidase IV inhibitor	1	0.333	0.091	0	0.000	0.000
8501	IP	dipeptidyl peptidase IV inhibitor	3	1.000	0.091	0	0.000	0.000
8637	AA	dipeptidyl peptidase IV inhibitor	1	0.333	0.091	0	0.000	0.000
8765	AY	dipeptidyl peptidase IV inhibitor	1	0.333	0.091	0	0.000	0.000
8858	PK	dipeptidyl peptidase IV inhibitor	1	0.333	0.091	0	0.000	0.000
8863	PT	dipeptidyl peptidase IV inhibitor	1	0.333	0.091	0	0.000	0.000
8914	TY	dipeptidyl peptidase IV inhibitor	1	0.333	0.091	0	0.000	0.000
8932	YA	dipeptidyl peptidase IV inhibitor	2	0.667	0.095	0	0.000	0.000

8938	YI	dipeptidyl peptidase IV inhibitor	3	1.000	0.091	0	0.000	0.000
9510	YI	dipeptidyl peptidase III inhibitor	3	1.000	0.091	0	0.000	0.000
9433	YA	renin inhibitor	2	0.667	0.095	0	0.000	0.000
3383	IY	ACE inhibitor	1	0.333	0.100	0	0.000	0.000
9072	DY	ACE inhibitor	1	0.333	0.100	0	0.000	0.000
2749	DY	regulating	1	0.333	0.100	0	0.000	0.000
7873	IY	antioxidative	1	0.333	0.100	0	0.000	0.000
8762	AS	dipeptidyl peptidase IV inhibitor	1	0.333	0.100	0	0.000	0.000
8812	KI	dipeptidyl peptidase IV inhibitor	2	0.667	0.091	0	0.000	0.000
3532	GY	ACE inhibitor	1	0.333	0.083	1	0.333	0.083
7543	AW	ACE inhibitor	1	0.333	0.083	0	0.000	0.000
7598	GA	ACE inhibitor	1	0.333	0.083	0	0.000	0.000
7613	WG	ACE inhibitor	1	0.333	0.083	0	0.000	0.000
7619	LG	ACE inhibitor	1	0.333	0.083	0	0.000	0.000
9079	IL	ACE inhibitor	1	0.333	0.083	0	0.000	0.000
8323	IL	stimulating	1	0.333	0.083	0	0.000	0.000
8460	AW	antioxidative	1	0.333	0.083	0	0.000	0.000
9082	WG	antioxidative	1	0.333	0.083	0	0.000	0.000
3201	GAW	inhibitor	1	0.333	0.083	1	0.333	0.083
8524	GA	dipeptidyl peptidase IV inhibitor	1	0.333	0.083	0	0.000	0.000
8676	WK	dipeptidyl peptidase IV inhibitor	1	0.333	0.083	0	0.000	0.000
8695	AW	dipeptidyl peptidase IV inhibitor	1	0.333	0.083	0	0.000	0.000
8697	WG	dipeptidyl peptidase IV inhibitor	1	0.333	0.083	0	0.000	0.000
8788	GY	dipeptidyl peptidase IV inhibitor	1	0.333	0.083	1	0.333	0.083
8802	IL	dipeptidyl peptidase IV inhibitor	1	0.333	0.083	0	0.000	0.000

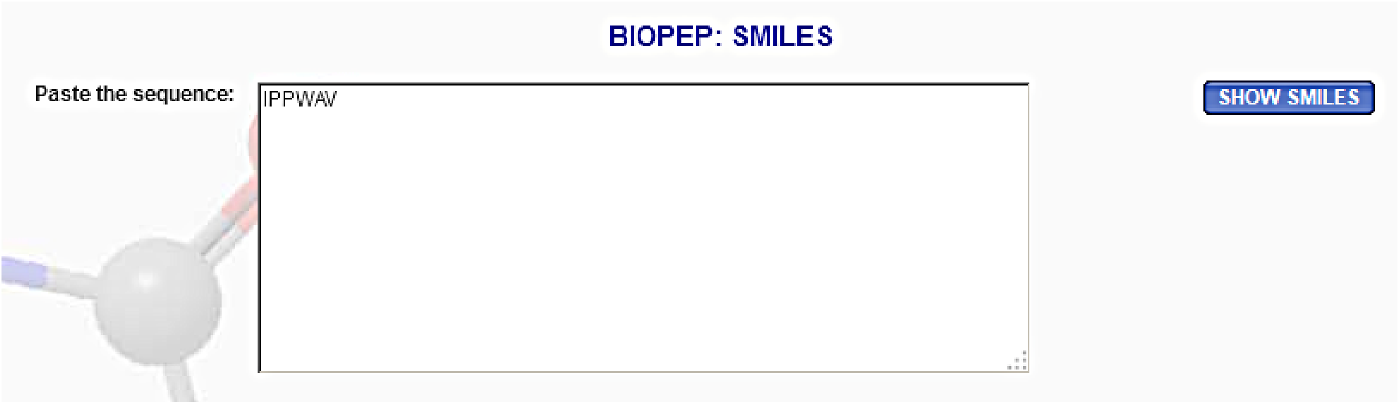

Figure S19. Screenshot of the “SMILES” tab. The “Show SMILES” button serves to display result.

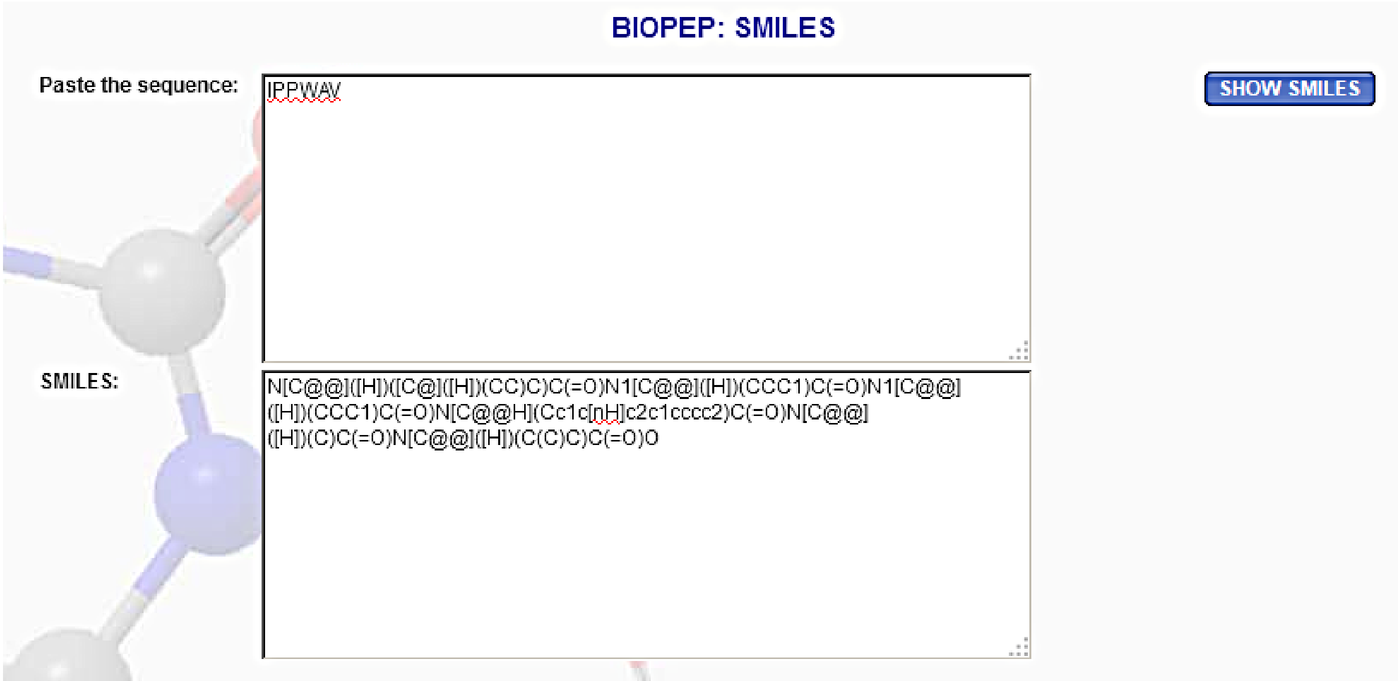

Figure S20. Screenshot of the "SMILES" tab with result of conversion of amino acid sequence of peptide IPPWAV into SMILES code.

Table S10. Two types of SMILES representations of peptide IPPWAV: aromatic version generated from the amino acid sequence by "SMILES" application in the BIOPEP-UWM database and Kekule version obtained from the previous one using molecule editor of the PubChem database

Kind of representation	Representation
Sequence	IPPWAV
Aromatic version of SMILES	N[C@@]([H])([C@]([H])(CC)C)C(=O)N1[C@@]([H])(CCC1)C(=O)N1[C@@]([H])(CCC1)C(=O)N[C@@H](Cc1c[nH]c2c1cccc2)C(=O)N[C@@]([H])(C)C(=O)N[C@@]([H])(C(C)C)C(=O)O
Kekule version of SMILES	N[C@@H]([C@H](CC)C)C(=O)N1[C@@H](CCC1)C(=O)N2[C@@H](CCC2)C(=O)N[C@@H](CC3=C[N]([H])C4=CC=CC=C34)C(=O)N[C@@H](C)C(=O)N[C@@H](C(C)C)C(=O)O

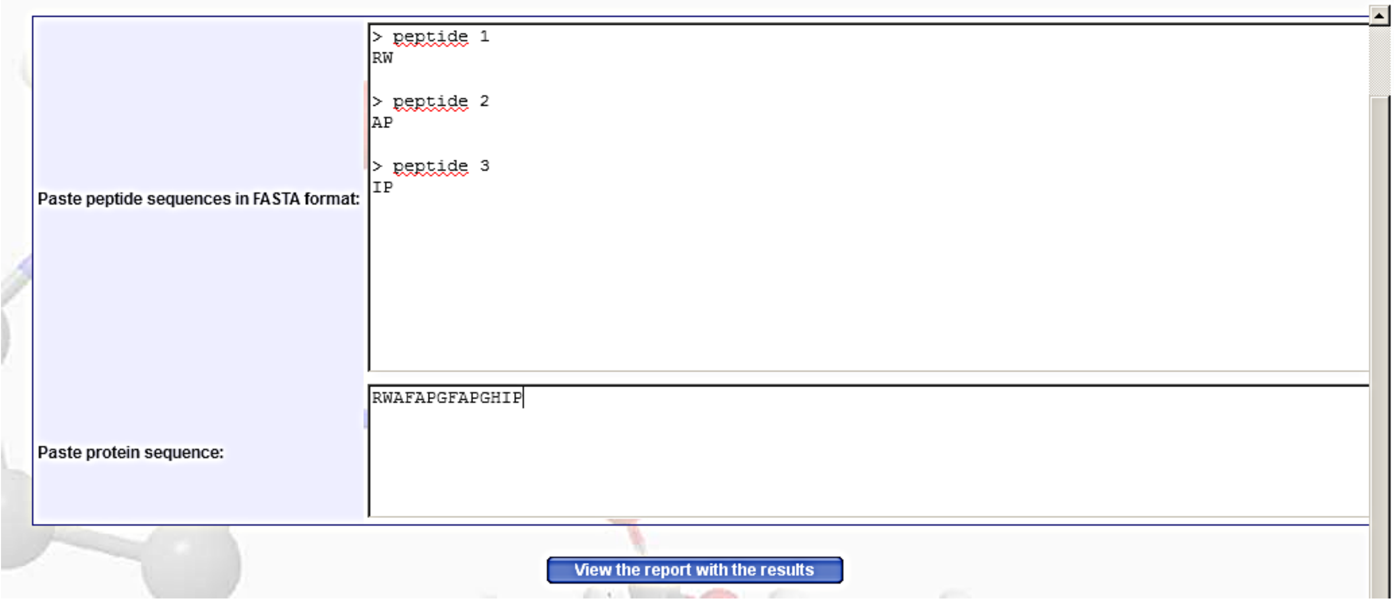

Figure S21. Screenshot of the menu of the "Find enzyme for peptide release" tab

Table S11. Results of the action of "Find enzyme for peptide release" option. Example covers prediction of release of peptides RW, AP, and IP from the RWAFAPGFAPGHIP sequence.

**Protein sequence**	**RWAFAPGFAPGHIP**				
**Peptide sequence**	**RW**				
**Location of peptide**	**[1-2]**				
**Location of proteolytic events**	**1C: 2-3**				
**Displaying of proteolytic events**	**RW-AFAPGFAPGHIP**				
Proteolytic event	***1C***				
Enzyme Name	EC Number	Enzyme ID	Connection ID	Cutting seqence	Recognition sequence
thermolysin	EC 3.4.24.27	18	49	A+	A
coccolysin	EC 3.4.24.30	40	133	A+	A
chymotrypsin (A)	EC 3.4.21.1	11	27	W-	W
Proteinase K	EC.3.4.21.67	14	39	W-	W
chymase	EC 3.4.21.39	23	64	W-	W
chymotrypsin C	EC 3.4.21.2	19	87	W-	W
metridin	EC 3.4.21.3	27	93	W-	W
subtilisin	EC 3.4.21.62	41	138	W-	W
**Protein sequence**	**RWAFAPGFAPGHIP**				
**Peptide sequence**	**AP**				
**Location of peptide**	**[5-6],[9-10]**				
**Location of proteolytic events**	**1N: 4-5; 1C: 6-7; 2N: 8-9; 2C: 10-11**				
**Displaying of proteolytic events**	**RWAF-AP-GF-AP-GHIP**				
Proteolytic event	***1N***				
Enzyme Name	EC Number	Enzyme ID	Connection ID	Cutting sequence	Recognition sequence
chymotrypsin (A)	EC 3.4.21.1	11	28	F-	F
pepsin (pH 1.3)	EC 3.4.23.1	13	32	F-	F
Proteinase K	EC.3.4.21.67	14	34	F-	F
cathepsin G	EC 3.4.21.20	21	58	F-	F
chymase	EC 3.4.21.39	23	62	F-	F
papain	EC 3.4.22.2	24	66	F-	F
ficin	EC 3.4.22.3	25	73	F-	F
metridin	EC 3.4.21.3	27	91	F-	F
pancreatic elastase II	EC 3.4.21.71	29	101	F-	F
oligopeptidase F	-	36	114	F-	F
subtilisin	EC 3.4.21.62	41	135	F-	F
thermolysin	EC 3.4.24.27	18	49	A+	A
papain	EC 3.4.22.2	24	71	A+	A
calpain 2	EC 3.4.22.53	33	109	A+	A
coccolysin	EC 3.4.24.30	40	133	A+	A

Proteolytic event	1C				
Enzyme Name	EC Number	Enzyme ID	Connection ID	Cutting sequence	Recognition sequence
Proteinase K	EC.3.4.21.67	14	40	P-	P
prolyl oligopeptidase	EC 3.4.21.26	16	47	P-	P
chymotrypsin C	EC 3.4.21.2	19	85	P-	P

Proteolytic event	2N				
Enzyme Name	EC Number	Enzyme ID	Connection ID	Cutting sequence	Recognition sequence
chymotrypsin (A)	EC 3.4.21.1	11	28	F-	F
pepsin (pH 1.3)	EC 3.4.23.1	13	32	F-	F
Proteinase K	EC.3.4.21.67	14	34	F-	F
cathepsin G	EC 3.4.21.20	21	58	F-	F
chymase	EC 3.4.21.39	23	62	F-	F
papain	EC 3.4.22.2	24	66	F-	F
ficin	EC 3.4.22.3	25	73	F-	F
metridin	EC 3.4.21.3	27	91	F-	F
pancreatic elastase II	EC 3.4.21.71	29	101	F-	F
oligopeptidase F	-	36	114	F-	F
subtilisin	EC 3.4.21.62	41	135	F-	F
thermolysin	EC 3.4.24.27	18	49	A+	A
papain	EC 3.4.22.2	24	71	A+	A
calpain 2	EC 3.4.22.53	33	109	A+	A
coccolysin	EC 3.4.24.30	40	133	A+	A

Proteolytic event	2C				
Enzyme Name	EC Number	Enzyme ID	Connection ID	Cutting seqence	Recognition sequence
Proteinase K	EC.3.4.21.67	14	40	P-	P
prolyl oligopeptidase	EC 3.4.21.26	16	47	P-	P
chymotrypsin C	EC 3.4.21.2	19	85	P-	P

Protein sequence	RWAFAPGFAPGHIP
Peptide sequence	IP
Location of peptide	[13-14]
Location of proteolytic events	1N: 12-13
Displaying of proteolytic events	RWAFAPGFAPGH-IP

Proteolytic event	1N				
Enzyme Name	EC Number	Enzyme ID	Connection ID	Cutting sequence	Recognition sequence
cathepsin G	EC 3.4.21.20	21	57	H-	H
chymotrypsin (A)	EC 3.4.21.1	11	142	H-	H
ficin	EC 3.4.22.3	25	185	H-	H

thermolysin	EC 3.4.24.27	18	51	I+	I
coccolysin	EC 3.4.24.30	40	175	I+	I
pepsin (pH > 2)	EC 3.4.23.1	39	189	I+	I
